# Immunotolerant p50/NFκB Signaling and Attenuated Hepatic IFNβ Expression Increases Neonatal Sensitivity to Endotoxemia

**DOI:** 10.3389/fimmu.2018.02210

**Published:** 2018-09-26

**Authors:** Sarah McKenna, Taylor Burey, Jeryl Sandoval, Leanna Nguyen, Odalis Castro, Suma Gudipati, Jazmin Gonzalez, Karim C. El Kasmi, Clyde J. Wright

**Affiliations:** Section of Neonatology, Department of Pediatrics, University of Colorado School of Medicine, Aurora, CO, United States

**Keywords:** neonate, endotoxemia, interferon beta, IRF3, STAT-1, NF-kappa B

## Abstract

Sepsis is a major cause of neonatal morbidity and mortality. The current paradigm suggests that neonatal susceptibility to infection is explained by an innate immune response that is functionally immature. Recent studies in adults have questioned a therapeutic role for IFNβ in sepsis; however, the role of IFNβ in mediating neonatal sensitivity to sepsis is unknown. We evaluated the transcriptional regulation and expression of IFNβ in early neonatal (P0) and adult murine models of endotoxemia (IP LPS, 5 mg/kg). We found that hepatic, pulmonary, and serum IFNβ expression was significantly attenuated in endotoxemic neonates when compared to similarly exposed adults. Furthermore, endotoxemia induced hepatic p65/NFκB and IRF3 activation exclusively in adults. In contrast, endotoxemia induced immunotolerant p50/NFκB signaling in neonatal mice without evidence of IRF3 activation. Consistent with impaired IFNβ expression and attenuated circulating serum levels, neonatal pulmonary STAT1 signaling and target gene expression was significantly lower than adult levels. Using multiple *in vivo* approaches, the source of hepatic IFNβ expression in endotoxemic adult mice was determined to be the hepatic macrophage, and experiments in RAW 264.7 cells confirmed that LPS-induced IFNβ expression was NFκB dependent. Finally, treating neonatal mice with IFNβ 2 h after endotoxemia stimulated pulmonary STAT1 signaling and STAT1 dependent gene expression. Furthermore, IFNβ treatment of endotoxemic neonatal animals resulted in significantly improved survival following exposure to lethal endotoxemia. In conclusion, endotoxemia induced IFNβ expression is attenuated in the early neonatal period, secondary to impaired NFκB-p65/IRF3 signaling. Pre-treatment with IFNβ decreases neonatal sensitivity to endotoxemia. These results support further study of the role of impaired IFNβ expression and neonatal sensitivity to sepsis.

## Introduction

Worldwide, sepsis is a leading killer of neonates (1). The current paradigm suggests that neonatal susceptibility to infection is explained by an innate immune response that is functionally immature, limited in its ability to mount efficient response, and “biased against the production of pro-inflammatory cytokines” (2–9). Thus, understanding the mechanisms that contribute to impaired production of the mediators of the innate immune response may reveal therapeutic targets meant to improve the outcomes of septic neonates.

The role of IFNβ in the pathogenesis of sepsis in adults is controversial. Produced by most nucleated cells, IFNβ ultimately activates immune cells, cytokine/chemokine production, and links the early innate and later adaptive immune response (10). It is well established that in adult murine models of endotoxemic shock, the transcription factors NFκB and IRF3 work together to induce IFNβ expression (11). IRF3 null, IFNβ null, IFN-α/β receptor (IFNAR) null, STAT1 null, and pharmacologic inhibition of the IFNAR improve protect adult mice from mortality with endotoxemic shock (12–15). Importantly, attenuating IFNβ activity has been proposed as a potential therapeutic target to treat in endotoxemia in experimental animals and in sepsis in humans (11).

In contrast to these findings, some experimental data support a protective role played by IFNβ in endotoxemia and sepsis. Type 1 IFN expression is required to limit viral infections, and its activation results in multiple anti-bacterial effects (16). Absent Type 1 interferon signaling increases mortality in adult murine polymicrobial sepsis (13). Importantly, downregulation of IFNβ has been implicated the period of immunosupression following the acute pro-inflammatory period of sepsis (17). Specifically, monocytes from immunosuppressed septic patients demonstrate attenuated IFNβ expression (18). These findings have led some to propose treating septic patients with IFNβ to restore the deactivated immune response (19).

It has been hypothesized that there may be common mechanisms underlying innate immune tolerance and the “developmentally immature immune response” that contributes to increased mortality in pediatric sepsis. If that were true, linking the mechanisms underlying impaired innate immune response and tolerance may reveal therapeutic targets to treat neonatal and pediatric sepsis. Altered signaling dynamics of the transcription factor NFκB have been implicated in mediating macrophage tolerance. Specifically, following TLR4 stimulation, tolerant macrophages demonstrate nuclear translocation of inhibitory p50 homodimers (20). Importantly, downregulation of LPS-induced IFN expression is mediated by transition from activating p65/p50 NFκB dimers to inhibitory p50 homodimers at the IFNβ promoter (18). Of note, LPS-induced IFNβ expression is impaired in neonatal blood (21). However, whether tolerant p50 dominant NFκB signaling results in impaired IFNβ expression and contributes to worse neonatal and pediatric outcomes in endotoxemia and sepsis is unknown.

Therefore, we hypothesized that the increased mortality seen in endotoxemic neonatal mice is due in part to impaired IFNβ expression. Furthermore, we hypothesized that similar to tolerant macrophages, that predominant inhibitory p50 NFκB signaling would underlie impaired IFNβ expression. In this study, we found significantly attenuated expression of hepatic IFNβ in endotoxemia neonatal mice when compared to similarly exposed adults. In the neonatal liver, this was associated with exclusive p50-NFκB activation, whereas the adult liver demonstrated nuclear translocation of both p50, p65, and p-IRF3. As evidence of impaired IFNβ expression in neonatal mice, we found impaired pulmonary STAT1 signaling and gene expression. Finally, treating endotoxemic neonatal mice with IFNβ restored pulmonary STAT1 signaling, gene expression and significantly decreased mortality. These results justify further investigation into the role of IFNβ in treating neonatal and pediatric sepsis.

## Materials and methods

### Murine model of endotoxemia

Neonatal (P0) and adult (8–10 weeks, male) ICR mice were exposed to LPS (Sigma L2630, 5 mg/kg, IP) for 0–24 h. Additional neonatal mice were treated with IFNβ (R and D Systems 8234-MB/CF, 0–100 U/g, IP) 2 h after a lethal dose of LPS (10 mg/kg, IP). Intrahepatic leukocytes were isolated, and hepatic macrophages were ablated with clodronate as previously described to assess their role in IFNβ production (22). All procedures were approved by the IACUC at the University of Colorado (Aurora, CO) and care and handling of the animals was in accord with the National Institutes of Health guidelines for ethical animal treatment.

### Cell culture, exposures, and pharmacologic NFκB inhibition

RAW 264.7 murine macrophages (ATCC) were cultured according to the manufacturer's instructions. Cells were exposed to LPS (1 μg/ml, Sigma L6529) or Interferon-β (100–1,000 U/ml, R and D systems). To pharmacologically inhibit NFκB activation, cells were exposed to BAY 11-7085 (1–20 μM, Sigma) for 1 h prior to LPS exposure.

### IκBα overexpression

RAW 264.7 cells were transfected with wild-type IκBα vectors (Clontech) as previously described (22).

### Pulmonary lysate, cytosolic, and nuclear protein extraction

Pulmonary tissue was homogenized using the Bullet Blender (NextAdvance) and pulmonary whole cell lysates were collected in T-PER (Thermo Fisher Scientific). Cytosolic and nuclear extracts were prepared using the NE-PER kit (ThermoFisher Scientific).

### Immunoblot analysis

Lysates, cytosolic, and nuclear extracts were electrophoresed on a 4–12% polyacrylamide gel (Invitrogen) and proteins were transferred to an Immobilon membrane (Millipore) and blotted with antibodies (Supplementary Table 1). Blots were imaged using the LiCor Odyssey imaging system and densitometric analysis was performed using ImageStudio (LiCor). Full blot images are found in Supplementary Figures 1–5.

### Analysis of relative mRNA levels by RT-qPCR

Pulmonary mRNA was collected using the RNeasy Mini Kit (Qiagen) according to the manufacturer's instructions. RNA was assessed for purity and concentration using the NanoDrop (ThermoFisher Scientific), and cDNA synthesized using the Verso cDNA synthesis Kit (ThermoFisher Scientific). Relative mRNA levels were evaluated by quantitative real-time PCR using exon spanning primers (Supplementary Table 2) and the TaqMan gene expression and StepOnePlus Real-Time PCR System (Applied Biosystems). Relative quantitation was performed via normalization to the endogenous control 18S using the cycle threshold (ΔΔCt) method.

### ELISA

Neonatal and adult serum levels of IFNβ were measured by ELISA (PBL Assay Science).

### Statistical analysis

For comparison between treatment groups, the null hypothesis that no difference existed between treatment means was tested by Student's *t*-test for two groups and two-way ANOVA for multiple groups with potentially interacting variables (organ, age, duration of exposure), with statistical significance between and within groups determined by means of Bonferroni method of multiple comparisons (InStat, GraphPad Software, Inc.,). Statistical significance was defined as *p* < 0.05.

## Results

### Endotoxemia induces hepatic IFNβ expression in adult but not neonatal mice

**Figure 1:**
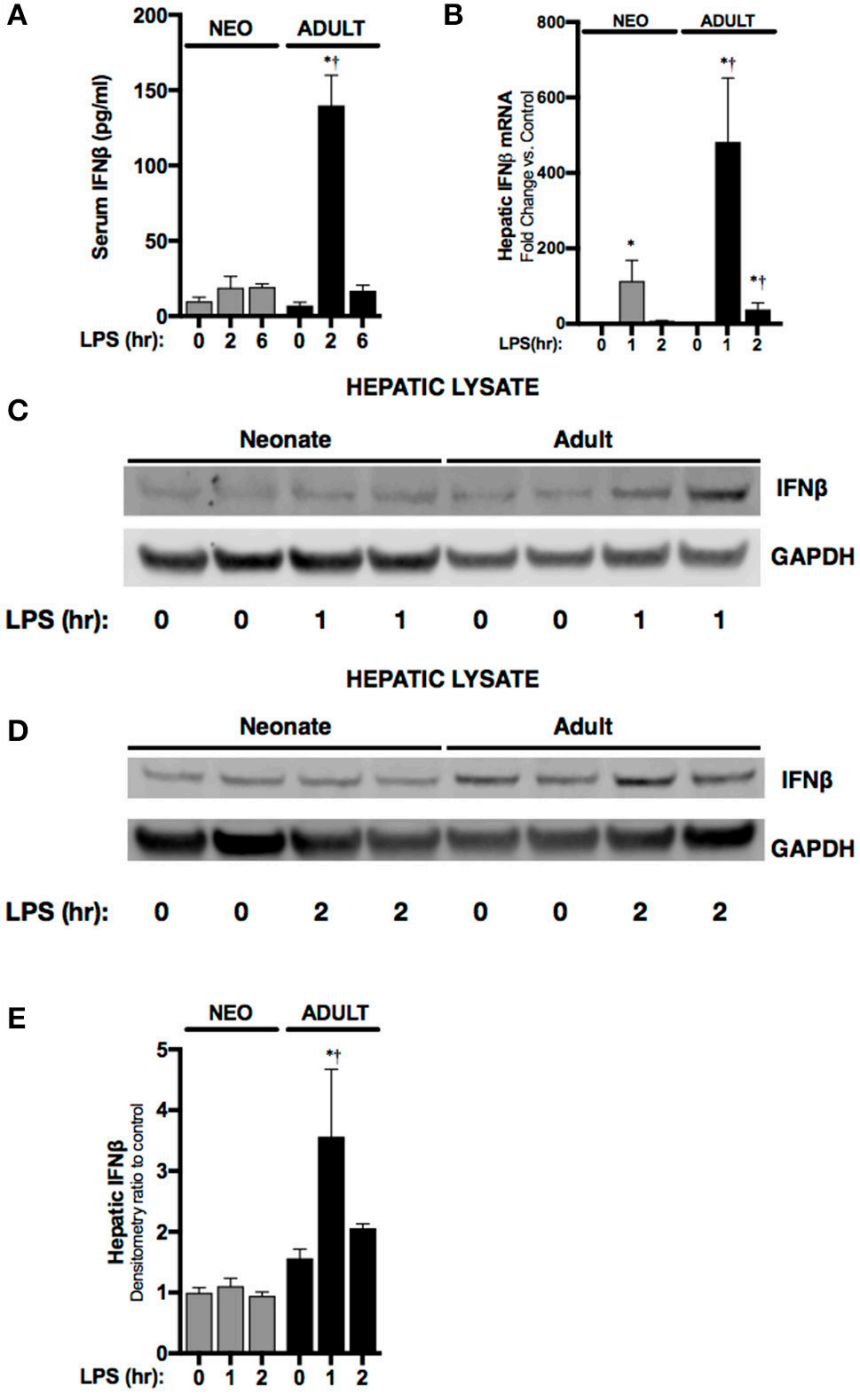
Neonates demonstrate attenuated LPS-induced hepatic IFNβ expression. **(A)** Serum levels of IFNβ in neonatal or adult mice following LPS exposure (0–6 h, 5 mg/kg). *p < 0.05 vs. control; †*p* < 0.05 vs. LPS-exposed neonate. Values shown as means ± SEM; *n* = 3–4/timepoint. **(B)** Fold-increase in gene expression of IFNβ in neonatal and adult liver following LPS exposure (0–2 h, 5 mg/kg). *p < 0.05 vs. control;†*p* < 0.05 vs. LPS-exposed neonate. Values shown as means ± SEM; *n* = 5–6/timepoint. **(C,D)** Representative Western blots showing IFNβ protein in neonatal and adult hepatic lysate following 1 h **(C)** or 2 h **(D)** of LPS exposure (5 mg/kg) with GAPDH shown as loading control. **(E)** Densitometry ratio to control of neonatal and adult hepatic IFNβ. *p < 0.05 vs. control; †*p* < 0.05 vs. LPS-exposed neonate. Values shown as means ± SEM; *n* = 2–3/timepoint.

First, we sought to determine whether endotoxemia induced IFNβ expression in neonatal mice. Consistent with previous reports, levels of circulating IFNβ were significantly higher in endotoxemic adult mice when compared to controls (Figure 1A). Following IP injection, LPS enters the portal circulation and stimulates hepatic macrophages (23). Thus, we assessed whether hepatic IFNβ expression increased with endotoxemia. Hepatic IFNβ mRNA expression significantly increased in both neonatal and adult liver after 1 h of endotoxemia (Figure 1B). However, at this time point, adult hepatic IFNβ mRNA induction was significantly higher compared to neonatal mice (Figure 1B). Consistent with impaired induction of IFNβ in endotoxemic neonatal mice, hepatic IFNβ protein expression (Figures 1C,D) and circulating serum levels (Figure 1A) were not significantly increased at this early time point in neonatal mice. In contrast, both hepatic protein (Figures 1C–E) and circulating serum levels (Figure 1A) were significantly increased in endotoxemic adult mice (Figures 1C–E). These results demonstrate that in contrast to observations made in adult mice, circulating IFNβ levels and hepatic IFNβ protein do not increase in endotoxemic neonatal mice.

### Endotoxemia induces hepatic IRF3 activity in adult but not neonatal mice

**Figure 2:**
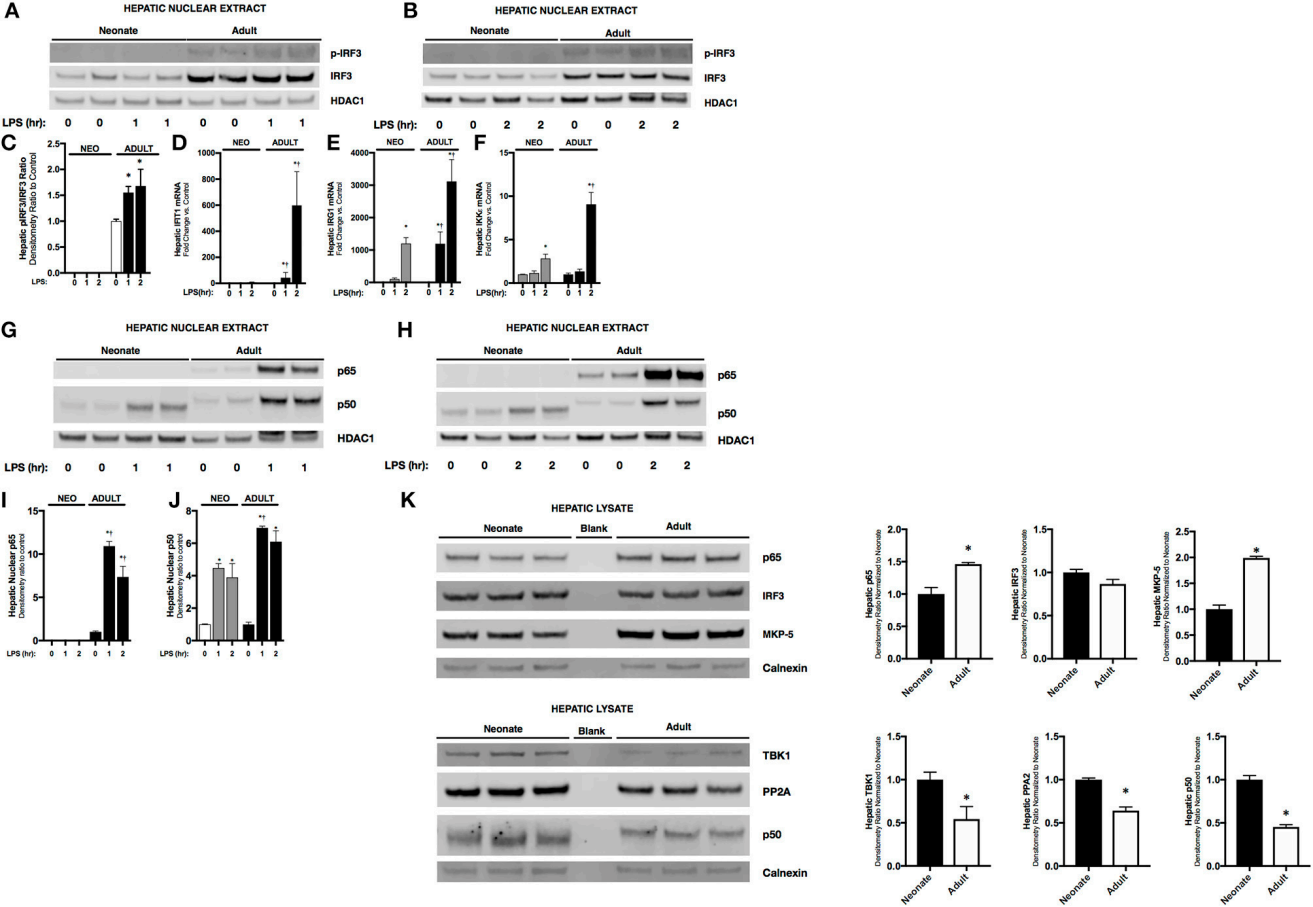
Neonates demonstrate attenuated LPS-induced hepatic IRF3 and p50/NFκB activation. **(A,B)** Representative Western Blot showing neonatal and adult hepatic nuclear extracts following 1 h **(A)** or 2 h **(B)** of LPS exposure (5 mg/kg) for phosphorylated and total IRF3 with HDAC1 shown as loading control. **(C)** Densitometry ratio to control of adult hepatic pIRF3. *p < 0.05 vs. control. Values shown as means ± SEM; *n* = 3/timepoint **(D–F)** Fold-increase in gene expression in neonatal and adult hepatic IFIT1, IRG1, and IKKε following LPS exposure (0–2 h, 5 mg/kg) *p < 0.05 vs. control; †*p* < 0.05 vs. LPS-exposed neonate. Values shown as means ± SEM; *n* = 3–6/timepoint. **(G, H)** Representative Western Blots showing neonatal and adult hepatic nuclear extract following 1 h **(G)** or 2 h **(H)** LPS exposure (5 mg/kg) for NFκB subunits p65 and p50 with HDAC1 shown as loading control. **(I,J)** Densitometry ratio to control of p65 or p50 in neonatal and adult hepatic nuclear extract following LPS exposure (0–2 h). *p < 0.05 vs. control; †*p* < 0.05 vs. LPS-exposed neonate. Values shown as means ± SEM; *n* = 5–6/timepoint. **(K)** Representative Western Blots showing neonatal and adult hepatic whole cell lysate for total IRF3 and the NFκB subunits p65 and p50, the kinase TBK1, and the IRF3 phosphatases PP2A catalytic subunit and MKP-5, with calnexin shown as loading control. Densitometry ratio normalized to neonatal control is provided. *p < 0.05 vs. neonatal control.

Having observed attenuated IFNβ expression in endotoxemic neonatal mice, we next investigated its transcriptional regulation. The transcription factor IRF3 is a known inducer of IFNβ expression. Importantly, IRF3 is expressed at easily detectable levels in both the neonatal and adult liver (Figure 2K). In the nuclear extracts isolated from endotoxemic adult mice, we observed significant increases in p-IRF3 (Figures 2A,B). This was associated with increased expression of IRF3 dependent genes IFIT1 (Figure 2C) and IRG1 (Figure 2D). Furthermore, hepatic expression of IKKε, the kinase responsible for phosphorylating and activating IRF3, was significantly increased in endotoxemic adult mice (Figure 2E). In contrast, we found evidence of absent or attenuated hepatic IRF3 activation in endotoxemic neonatal mice. Hepatic nuclear extracts isolated from endotoxemic neonatal mice did not demonstrate presence of p-IRF3 (Figures 2A,B), and expression of IRF3 dependent genes was variably absent (IFIT1, Figure 2D) or attenuated compared to adult mice (IRG1, Figure 2E). Of note, hepatic expression of the activating kinase IKKε was also attenuated in endotoxemic neonatal mice (Figure 2F). Furthermore, we could not detect decreased levels of the IRF3 kinase TBK1, or increased levels of the IRF3 phosphatases PP2A (24) and MKP-5 (25), findings that if present may help explain the mechanisms underlying lack of p-IRF3 in the LPS-exposed neonatal hepatic nuclear extracts (Figure 2K). These results demonstrate that activation of the transcription factor responsible for IFNβ expression is attenuated in endotoxemic neonatal mice.

### Endotoxemia induces hepatic p65 and p50 nuclear translocation in adults, and exclusively p50 in neonatal mice

Previous studies have shown that downstream of TLR4 stimulation, IFNβ upregulation is dependent upon both IRF3 and the NFκB dimers containing the subunit p65 (26). Thus, we sought to determine whether there were differences between NFκB subunits in hepatic nuclear extracts isolated from endotoxemic neonatal and adult mice. Importantly, p65 is expressed in both the neonatal and adult liver, although levels in the adult liver are significantly higher (Figure 2K). Furthermore, p50 is expressed in both the neonatal and adult liver, however levels in the neonatal liver are significantly higher (Figure 2K). Interestingly, we found that hepatic NFκB signaling was distinct in endotoxemic neonatal and adult mice. In endotoxemic adult mice, there was nuclear translocation of p65 and p50 at 1 and 2 h of exposure (Figures 2G–J). In contrast, no p65 nuclear translocation was observed in endotoxemic neonatal mice (Figures 2G–I). However, we did observe significant nuclear translocation of p50 in endotoxemic neonatal mice (Figures 2G,H,J). These results suggest that the impaired IFNβ expression observed in endotoxemic neonatal mice is due to absence of both nuclear p-IRF3 (Figures 2A,B) and p65 (Figures 2F–H).

### LPS-induced IFNβ expression in macrophages is NFκB regulated

**Figure 3:**
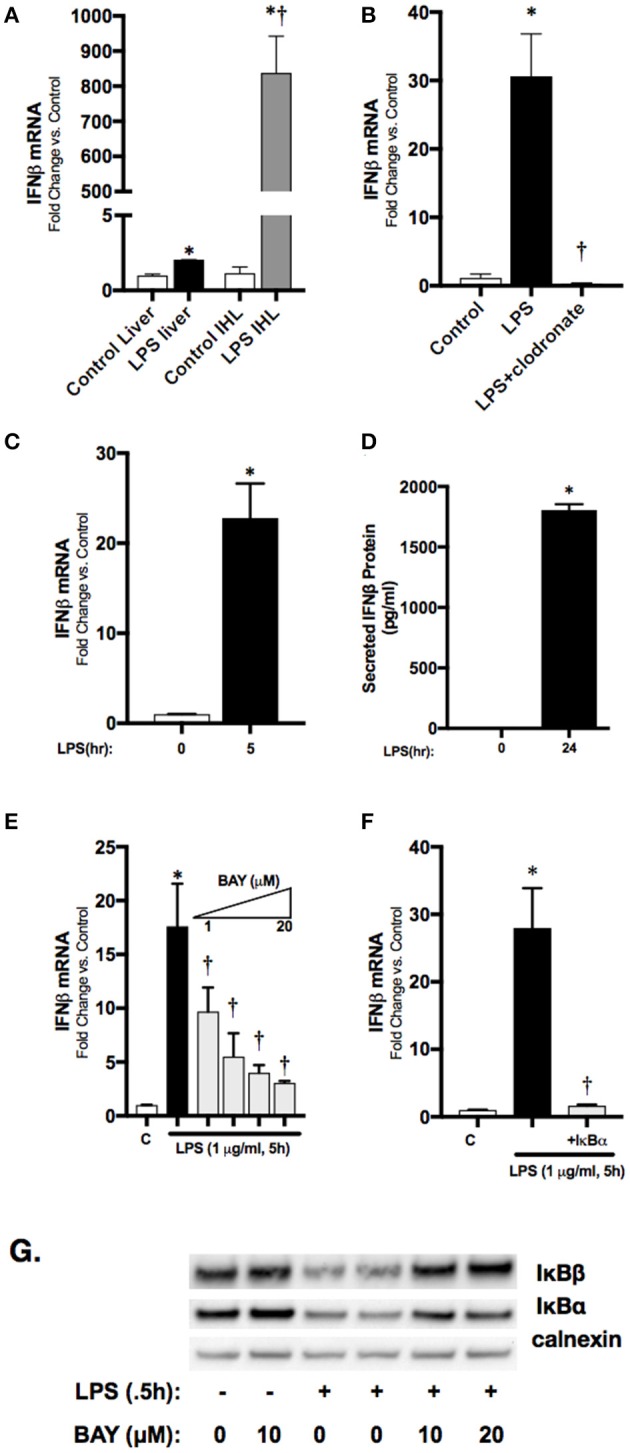
Macrophage-derived, LPS-induced IFNβ stimulates STAT1 activation *in vitro***. (A)** Fold-increase in gene expression of IFNβ in adult whole liver or isolated intrahepatic leukocytes following LPS exposure (2 h, 50 mg/kg). *p < 0.05 vs. control; †*p* < 0.05 vs. LPS-exposed whole liver. Values shown as means ± SEM; *n* = 4–6/timepoint. **(B)** Fold-increase in gene expression of IFNβ in adult liver following LPS exposure or clodronate pretreatment (24 h) with LPS exposure (4 h, 3 mg/kg). *p < 0.05 vs. control; †*p* < 0.05 vs. LPS-exposed. Values shown as means ± SEM; *n* = 4–6/timepoint **(C)** Fold-increase in gene expression of IFNβ in RAW 264.7 macrophages following LPS exposure (0–5 h, 1 μg/ml). *p < 0.05 vs. control. Values shown as means ± SEM; *n* = 4/timepoint **(D)** Secreted IFNβ protein measurement in RAW 264.7 culture supernatant following LPS exposure (0–24 h, 1 μg/ml). *p < 0.05 vs. control. Values shown as means ± SEM; *n* = 4/timepoint **(E)** Fold-increase in gene expression of IFNβ in RAW 264.7 macrophages following LPS exposure (0–5 h, 1 μg/ml) or BAY 11–7085 pretreatment (1 h, 1–20 μM) and LPS exposure. *p < 0.05 vs. control; †*p* < 0.05 vs. LPS-exposed. Values shown as means ± SEM; *n* = 4/timepoint. **(F)** Fold-increase in gene expression of IFNβ following LPS exposure (1 μg/ml, 5 h) or IκBα overexpression and LPS exposure. *p < 0.05 vs. control; †*p* < 0.05 vs. LPS-exposed Values shown as means ± SEM; *n* = 4/timepoint. **(G)** Representative Western blot showing IκBα and IκBβ in cytosolic fractions following exposure to LPS (0.5 h), or LPS after pretreatment (1 h) with BAY 11–7085 (1–20 μM) with calnexin as loading control.

To localize hepatic IFNβ expression, we determined IFNβ mRNA expression in purified intrahepatic mononuclear cells (ihMNCs) isolated from livers of endotoxemic adult mice. This population of ihMNCs is inclusive of macrophage populations (27). Compared to the significant ~2-fold increased IFNβ expression in whole liver from LPS-exposed mice, expression of IFNβ in ihMNCs was increased ~800-fold compared to ihMNCs from untreated mice (Figure 3A). In addition, clodronate-mediated ablation of hepatic macrophages completely abrogated LPS-induced hepatic IFNβ expression (Figure 3B). These results identify hepatic macrophages as a potential source of circulating IFNβ observed in endotoxemic adult mice.

Having identified the hepatic macrophage as a potential source of circulating IFNβ, we next sought to link LPS-induced NFκB signaling to IFNβ expression in macrophages. For these *in vitro* experiments we used immortalized murine macrophages (RAW 264.7). In cultured RAW 264.7 cells, LPS induced significant expression of IFNβ mRNA by 5 h of expression (Figure 3C), and levels could be measured in the cell media at 24 h (Figure 3D). Our previous work has shown that LPS-induced p65 nuclear translocation occurs in RAW 264.7 cells by 2 h of exposure (22). To confirm that LPS-induced NFκB activation regulates IFNβ in RAW 264.7 macrophages, cells were pretreated with the pharmacologic NFκB inhibitor BAY 11–7085 for 1 h prior to LPS (1 μg/ml, 1 h) exposure. Pre-treatment with BAY 11–7085 inhibited LPS-induced degradation of the NFκB inhibitory proteins IκBα and IκBβ (Figure 3G), and inhibited expression the IFNβ in a dose-dependent manner (Figure 3E). To rule out off-target effects of BAY 11–7085 on IFNβ expression independent of NFκB signaling, we transfected RAW 264.7 cells with plasmids overexpressing wild-type (WT) IκBα. Following exposure to LPS, IFNβ expression was significantly attenuated in cells overexpressing the inhibitory protein WT IκBα (Figure 3F). These results implicate LPS-induced NFκB activation in the transcriptional regulation of IFNβ in macrophages.

### LPS-induced JAK/STAT signaling is impaired in the neonatal lung with endotoxemia

**Figure 4:**
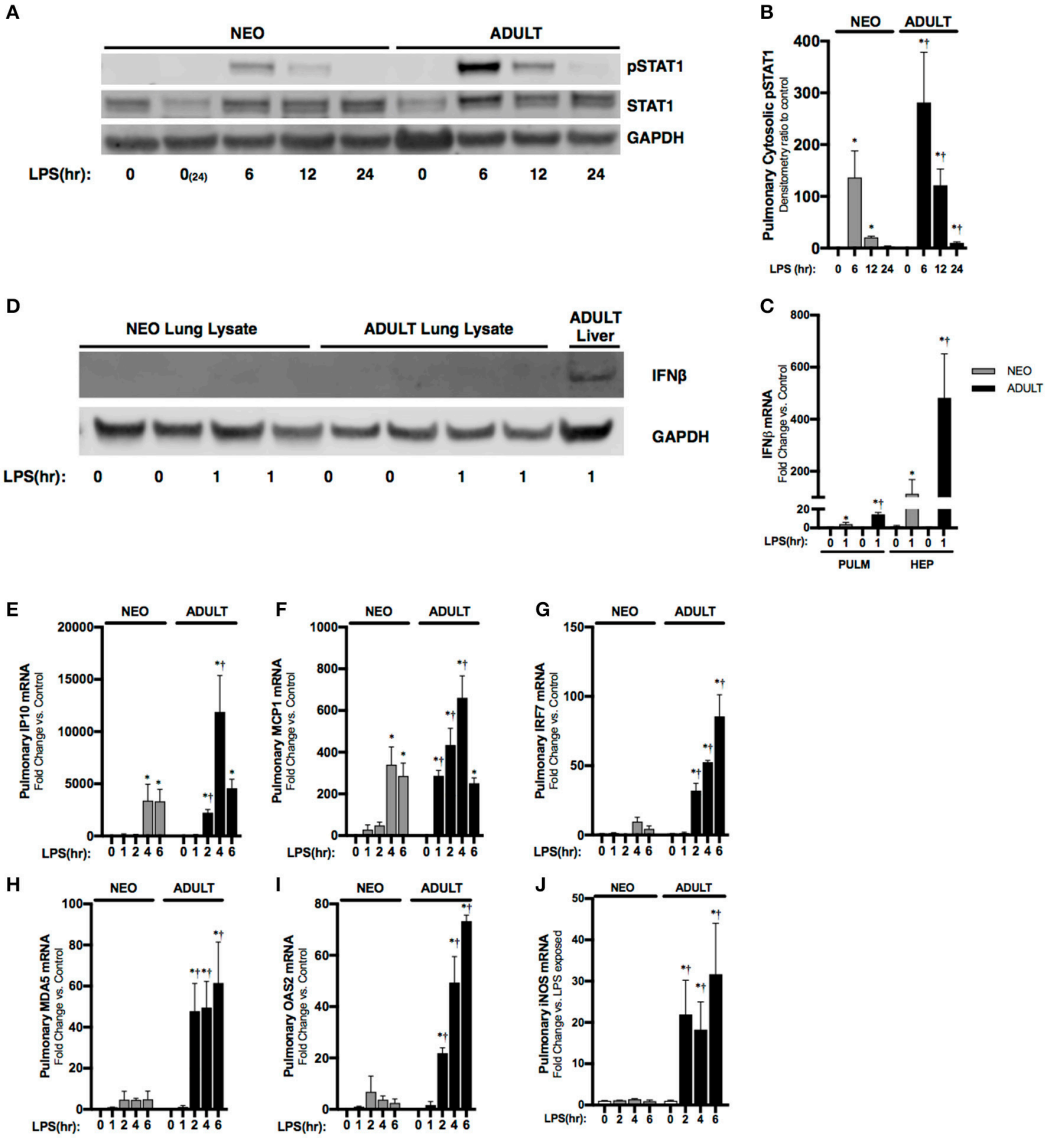
Neonates demonstrate attenuated LPS-induced pulmonary STAT1 activation downstream of hepatic IRF3 activation and IFNβ secretion. **(A)** Representative Western Blot of neonatal and adult pulmonary cytosolic extracts following LPS exposure (0–24 h, 5 mg/kg) for phosphorylated and total STAT1 with GAPDH shown as loading control; two neonatal controls shown to reflect baseline conditions on PN0 and after 24 h of life, at the completion of the exposure period. **(B)** Densitometry ratio to control of phosphorylated STAT1 in neonatal and adult pulmonary cytosolic extracts following LPS exposure. *p < 0.05 vs. control; †*p* < 0.05 vs. LPS-exposed neonate. Values shown as means ± SEM; *n* = 3–4/timepoint. **(C)** Fold-increase in gene expression of neonatal and adult pulmonary and hepatic IFNβ following LPS exposure (0–1 h, 5 mg/kg). *p < 0.05 vs. control; †*p* < 0.05 vs. LPS-exposed neonate. Values shown as means ± SEM; *n* = 5–6/timepoint. **(D)** Representative Western Blot of neonatal and adult pulmonary lysate following LPS exposure (0–1 h, 5 mg/kg) for IFNβ with adult LPS-exposed liver lysate provided as positive control and GAPDH shown as loading control. **(E–J)** Fold-increase in gene expression of STAT1 target genes **(E)** IP10, **(F)** MCP1, **(G)** IRF7, **(H)** MDA5, **(I)** OAS2, and **(J)** iNOS in neonatal and adult pulmonary tissue following LPS exposure (0–6 h, 5 mg/kg). *p < 0.05 vs. control; †*P* < 0.05 vs. LPS-exposed neonate. Values shown as means ± SEM; *n* = 5–6 per timepoint.

Next, we sought to understand the systemic implications of impaired hepatic IFNβ expression observed in neonatal mice. IFNβ is a known inducer of STAT1 activation (10), and pulmonary STAT1 activation is known to occur with endotoxemia (28). Consistent with previous reports, we found STAT1 phosphorylation in the pulmonary lysates of endotoxemic adult mice (Figures 4A,B). In contrast, STAT1 phosphorylation, while present, was attenuated in degree and duration in the lungs of endotoxemic neonatal mice (Figures 4A,B). It is likely that the hepatic derived IFNβ results in pulmonary STAT1 signaling, as LPS-induced hepatic IFNβ expression is significantly higher than pulmonary induction (Figure 4C). Additionally, we could not detect any IFNβ in pulmonary lysates from endotoxemic neonatal or adult mice (Figure 4D). These results demonstrate that in endotoxemic adult mice, hepatic IFNβ expression is temporally associated with pulmonary STAT1 signaling. This signaling is attenuated in duration and degree in endotoxemic neonatal mice.

### Stat dependent gene expression is attenuated in the neonatal lung with endotoxemia

Having observed attenuated STAT1 signaling in the neonatal lung, we next checked the expression of interferon stimulated genes previously shown to be dependent upon JAK/STAT signaling (29–31). Consistent with attenuated IFNβ-stimulated pulmonary STAT signaling, we found significantly attenuated expression of multiple STAT-dependent genes in the neonatal lung. These included IP10 (Figure 4E), MCP1 (Figure 4F), IRF7 (Figure 4G), MDA5 (Figure 4H), and OAS2 (Figure 4I). Furthermore, IFNβ/STAT1 activation are responsible for LPS-induced iNOS gene expression (32). Consistent with impaired LPS-induced STAT-1 signaling in the neonatal lung, we found significantly lower iNOS expression when compared to similarly exposed adults (Figure 4J).

### IFNβ treatment restores pulmonary STAT1 signaling and improves survival of endotoxemic neonatal mice

**Figure 5:**
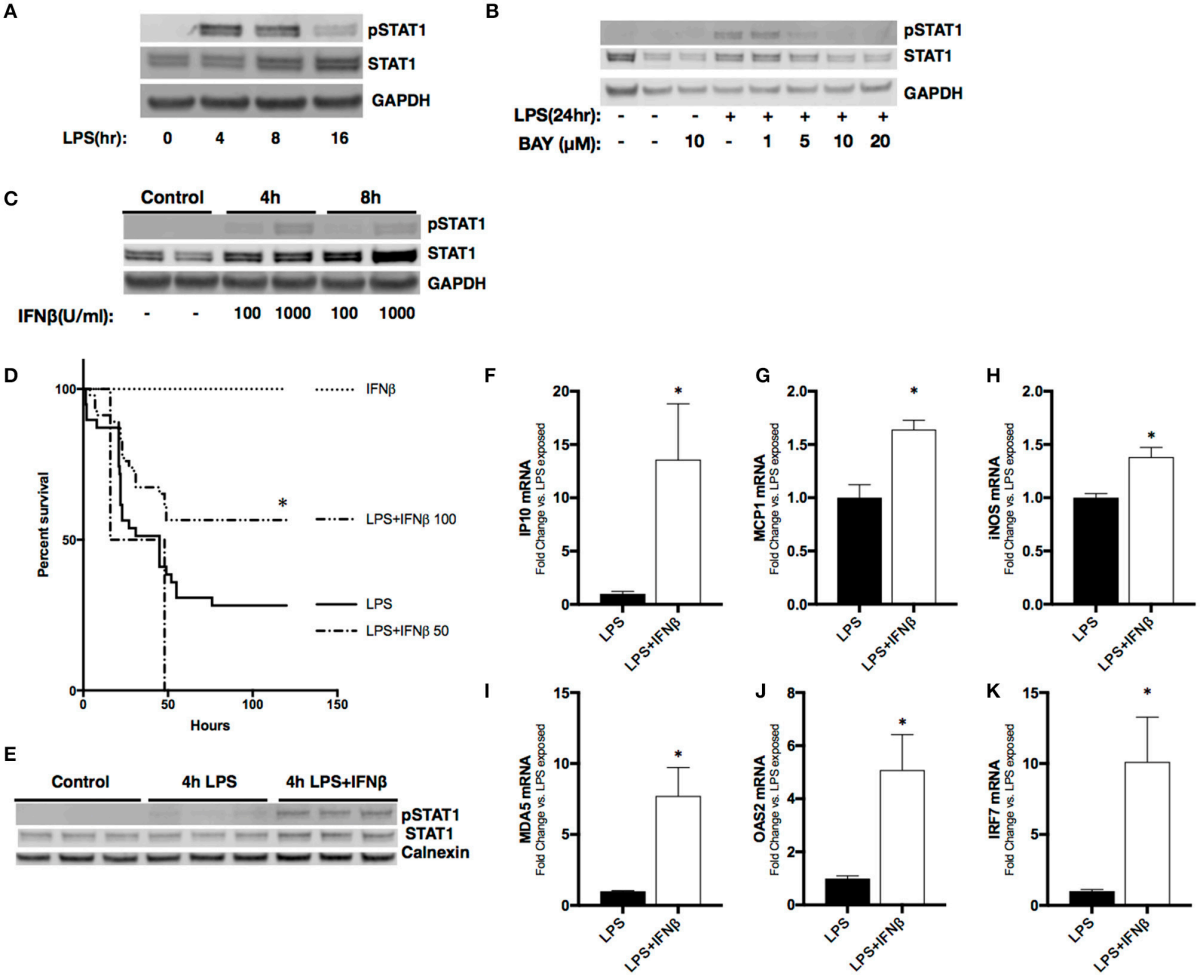
Treatment with IFNβ following LPS exposure reduces mortality and activates pulmonary STAT1 signaling in neonatal mice. **(A)** Representative Western Blot of phosphorylated and total STAT1 in RAW 264.7 macrophage lysate following LPS exposure (1 μg/ml, 0–16 h) with GAPDH shown as loading control. **(B)** Representative Western Blot of phosphorylated and total STAT1 in RAW 264.7 lysates following LPS exposure (1 μg/ml, 0–24 h) or BAY 11-7085 pretreatment (1–20 μM, 1 h) and LPS exposure with GAPDH shown as loading control. **(C)** Representative Western Blot of phosphorylated and total STAT1 in RAW 264.7 lysates following IFNβ exposure (100–1,000 μ/ml, 0–8 h) with GAPDH shown as loading control. **(D)** Kaplan-Meier curve analysis of neonatal mice exposed to IFNβ (100 μ/g; *n* = 7), LPS (10 mg/kg; *n* = 39), or LPS and IFNβ (50 (*n* = 7) or 100 μ/g (*n* = 46), administered 2 h post-LPS. *p < 0.05 vs. LPS-exposed; *n* = 7–40 per group. **(E)** Representative Western Blot of phosphorylated and total STAT1 in neonatal pulmonary lysates following LPS exposure (5 mg/kg, 4 h) or LPS exposure and IFNβ exposure (100 μ/g, administered 2 h post-LPS). *n* = 3/timepoint **(F–K)** Fold-increase in gene expression of STAT1 target genes IP10, MCP1, iNOS, OAS2, MDA5, and IRF7 in neonatal lung following LPS (5 mg/kg, 4 h) or LPS exposure and IFNβ exposure (100 μ/g, 2 h post-LPS). Values shown as means of fold induction normalized to mean LPS-induced fold induction ± SEM. *p < 0.05 vs. LPS-exposed; *n* = 3–5/timepoint.

Previous studies have demonstrated that absence of STAT1 activity exacerbates lung injury associated with endotoxemia (33, 34). We next sought to determine whether we could augment neonatal pulmonary STAT signaling by bypassing impaired LPS-induced hepatic IFNβ expression through direct administration of IFNβ after the induction of endotoxemia. First, we sought to determine if this was possible in cell culture. In RAW 264.7 cells, LPS induces STAT1 phosphorylation (Figure 5A). Importantly, LPS-induced STAT1 activation is completely inhibited by the NFκB inhibitor BAY 11–7085 (Figure 5B), likely due to impaired NFκB-regulated IFNβ expression (Figure 3E). We then demonstrated that exposing RAW 264.7 cells to IFNβ in the absence of LPS resulted in dose-dependent increase in STAT1 activation (Figure 5C). These results demonstrate macrophages respond directly to IFNβ with STAT1 signaling in the absence of LPS-TLR4 mediated NFκB activation.

Next, we sought to determine the effect of administering IFNβ to endotoxemic neonatal mice. Neonatal (P0) mice were exposed to endotoxemia (10 mg/kg) and experienced ~75% mortality (Figure 5D). When administered 2 h after LPS, IFNβ at a dose of 50 U/g had no effect on survival. In contrast, a dose of 100 U/g significantly improved survival to >50%. Importantly, administration of IFNβ after 2 h of endotoxemia induced pulmonary STAT1 signaling (Figure 5E). The IFNβ dependent induction of pulmonary STAT1 signaling significantly increased pulmonary expression of STAT1-dependent genes including IP10, MCP1, IRF7, OAS2, MDA5, and iNOS when compared to LPS alone (Figures 5F–K). These results demonstrate that in neonatal endotoxemia, IFNβ treatment improves survival and that this is associated with increased pulmonary STAT1 signaling and gene expression.

## Discussion

Our study revealed attenuated IFNβ expression in endotoxemic neonatal mice when compared to similarly exposed adults. In endotoxemic adult mice, activation of the NFκB-p65 and IRF3 transcription factors is associated with hepatic IFNβ expression, and occurs in the hepatic macrophage. In the setting of hepatic IFNβ expression in adult mice, pulmonary STAT1 signaling and increased expression of STAT1-dependent genes occurs. In contrast to these findings in adults, endotoxemic neonatal mice demonstrate attenuated hepatic IFNβ expression. We could find no evidence of hepatic IRF3 or p65-NFκB activation in endotoxemic neonatal mice. In contrast, hepatic p50-NFκB signaling was observed. In the absence of hepatic IFNβ expression, we observed attenuated pulmonary STAT1 signaling and target gene expression in neonatal mice. By treating endotoxemic neonatal mice with IFNβ, pulmonary STAT1 signaling was restored and this was associated with a significant decrease in mortality.

The type 1 response to endotoxemia and sepsis in adults has been an area of intense study and has been offered as a therapeutic target (11). Early reports showed that IRF3 null, IFNβ null, IFN-α/β receptor (IFNAR) null, STAT1 null, and pharmacologic inhibition of the IFNAR improve outcomes in endotoxemic adult mice (12–15). However, IFNβ affects the immune system via multiple mechanisms, such that it has been concluded that “IFN-1 are neither “good” nor “bad” regulators of inflammation, but that their protective or adverse character varies with more or less pronounce inflammatory environments.” (35) This may explain somewhat conflicting data in the literature regarding the role of interferons in mediating the response to endotoxemia and/or sepsis. For example, IFNAR1 mice have shown both increased resistance and sensitivity to polymicrobial sepsis (13, 15). Furthermore, recent reports have demonstrated that IFNβ may protect adult mice against lethal endotoxemia (36). These nuanced findings may have particular relevance in understanding the role of IFNβ in mediating the neonatal response to endotoxemia and sepsis.

Neonates and adults display markedly different susceptibilities to endotoxemia and sepsis. Neonatal animals (mice, rats, guinea pigs) demonstrate increased mortality when compared to adults following exposure to bacterial endotoxin shock (37–44). Multiple recent reviews of early life immunity conclude that this is in part due to an impaired ability to mount a pro-inflammatory innate immune response in the perinatal period (2, 4–6, 8, 9). Of note, previous studies have demonstrated that adult ICR mice demonstrate less sensitivity to endotoxemia when exposed to the exposure doses used in the current study (45, 46). Our work adds to a growing body of literature demonstrating that impaired IFNβ expression in the perinatal period contributes to these findings. *In vitro* work has showed that LPS-induced IFNβ expression is blunted in neonatal cord blood cells (21), and this observation is true following exposure to other TLR ligands (47). Of note, completely absent IFNβ signaling (IFNβ null and IFNα/β receptor null) in neonatal mice leads to 100% mortality following GBS infection (48) Furthermore, studies in adult mice demonstrate that IFNβ plays a protective role against infections common in the perinatal period, including Group B streptococcus and *E. coli* (49). Our report provides evidence that impaired LPS-induced IFNβ expression in the early neonatal period may contribute to increased susceptibility to certain infections.

Our results demonstrate that there are fundamental differences between neonatal and adult LPS-induced hepatic NFκB signaling and target gene expression. Previous studies have demonstrated that LPS/TLR4 mediated IRF3/ISRE activation is p65 dependent (26), and that IFNβ is an NFκB target gene (50). Our results clearly demonstrate a lack of p65/NFκB signaling in the neonatal liver following exposure to LPS (Figure 3). In contrast, the adult liver demonstrates robust p65/NFκB signaling and associated IRF3 activation (Figure 2) and IFNβ expression (Figure 1). Interestingly, the p50/NFκB signaling observed in the neonatal liver has been implicated in macrophage tolerance and M2 polarization (18, 20). Importantly, p50/NFκB signaling attenuates IFNβ expression and drives a macrophage tolerance and an M2 phenotype. Additionally, it should be noted that the p50 in neonatal hepatic nuclear extracts consistently migrates further when subjected to electrophoresis through a 4-12% polyacrylamide gel (Figures 2G,H). We hypothesize that this difference is due to post-translational modification of the p50 subunit in the adult liver. Of note, other groups have identified sites subject to post-translational modification on the p50 subunit (51). The implications of these potential modifications remain to be discovered. Further work is needed to understand whether these post-translational modifications explain our findings, and whether what we have observed in the neonatal endotoxemia model is a true “recapitulation of immune tolerance” (9).

This study has a number of limitations. Specifically, only one LPS dose (5 mg/kg, IP) was used for neonatal and adult endotoxin exposure; importantly, our lab has identified this as a dose that results in ~25% mortality in neonatal mice (52); thus, it is possible that alterations in IFNβ expression and STAT1 signaling might be observed at increasingly lethal LPS doses. Additionally, samples were collected at relatively early timepoints following LPS exposure, and there may be differences in neonatal and adult IFNβ expression at later time points. However, IFNβ is a primary response gene and previous publications have shown early and robust upregulation in endotoxemia (53). Our study did not specifically interrogate other tissues beyond the lung and liver as potential sources of IFNβ. However, LPS exposure results in widespread systemic effects, and significant IFNβ release may occur in other organs. However, we did assess circulating IFNβ levels in endotoxemic adult and neonatal mice, and regardless of expression in other organs, our observations in the liver and lung are valid. Finally, while treating endotoxemic neonatal mice with IFNβ resulted in pulmonary STAT1 activation and increased target gene expression, the direct mechanisms contributing to improved survival are unknown. Finally, *in vitro* experiments linking LPS-induced NFκB activity and IFNβ expression in macrophages were not performed in primary cell lines, but rather in immortalized murine macrophages (RAW 264.7, Figures 3C–H). We chose to use this cell line given the difficulty in transfecting primary cell lines and their susceptibility to pharmacologic agents.

## Conclusions

We conclude that LPS-induced hepatic IFNβ expression is attenuated in neonatal animals when compared to similarly exposed adults. Our data suggest that this is associated with LPS-induced p50-NFκB signaling and impaired IRF3 activation. These results are interesting known mechanistic role played by p50 in mediating macrophage phenotype and tolerance. Our findings support the hypothesis that in the neonatal period, there are shared mechanisms between an impaired innate immune response and immune tolerance. Treating endotoxemic neonatal mice with IFNβ restores pulmonary STAT1 signaling, STAT1 dependent gene expression and improves survival. These results justify further investigation into the role of both IFNβ and STAT1 signaling in treating neonatal and pediatric sepsis.

## Author contributions

CW and SM conception and design of research. SM, TB, JS, LN, OC, SG, JG, KE, and CW performed the experiments. SM, TB, JS, LN, OC, SG, JG, KE, and CW analyzed data; SM, TB, JS, OC, SG, JG, KE, and CW interpreted results of the experiments. SM and CW edited and revised the manuscript. SM, TB, JS, LN, OC, SG, JG, KE, and CW approved the final version of the manuscript. SM and CW prepared the figures. CW drafted the manuscript.

### Conflict of interest statement

The authors declare that the research was conducted in the absence of any commercial or financial relationships that could be construed as a potential conflict of interest.
