# Supplementary figures and images for: Immunotolerant p50/NFκB Signaling and Attenuated Hepatic IFNβ Expression Increases Neonatal Sensitivity to Endotoxemia
Source: Front Immunol. 2018 Sep 26;9:2210. doi: 10.3389/fimmu.2018.02210 (PMC6168645; doi:10.3389/fimmu.2018.02210)

Fig 1A

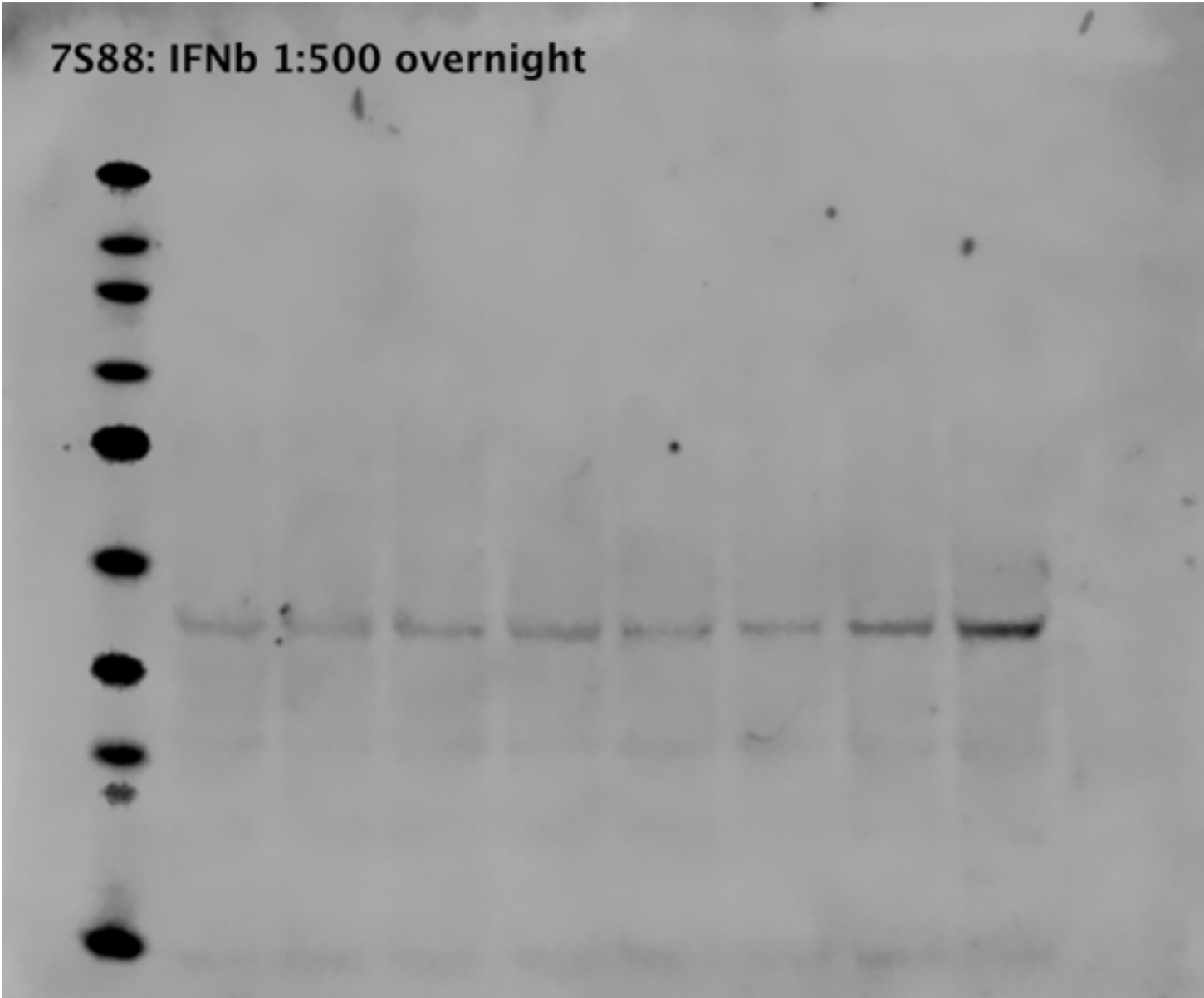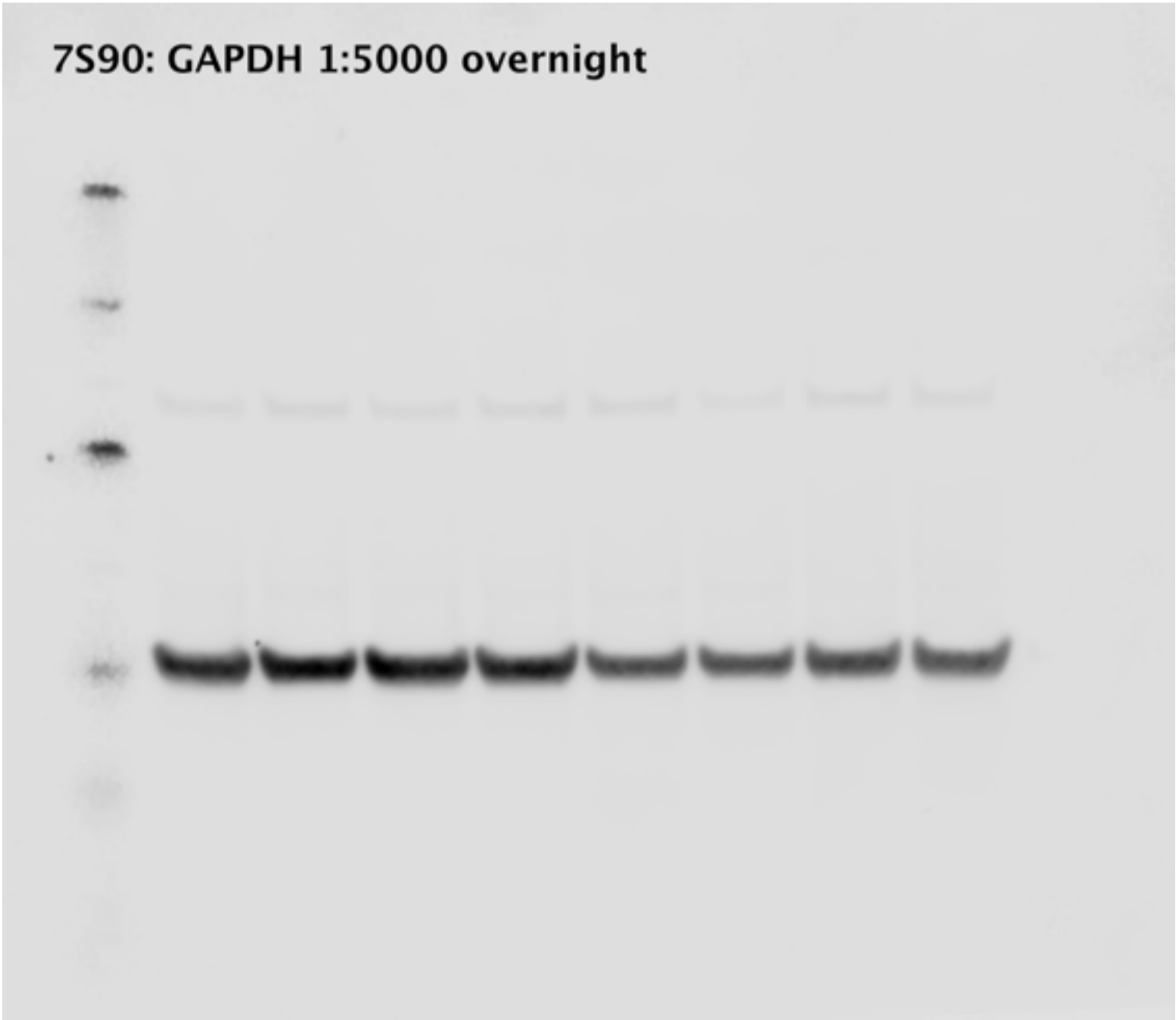

Fig 1B

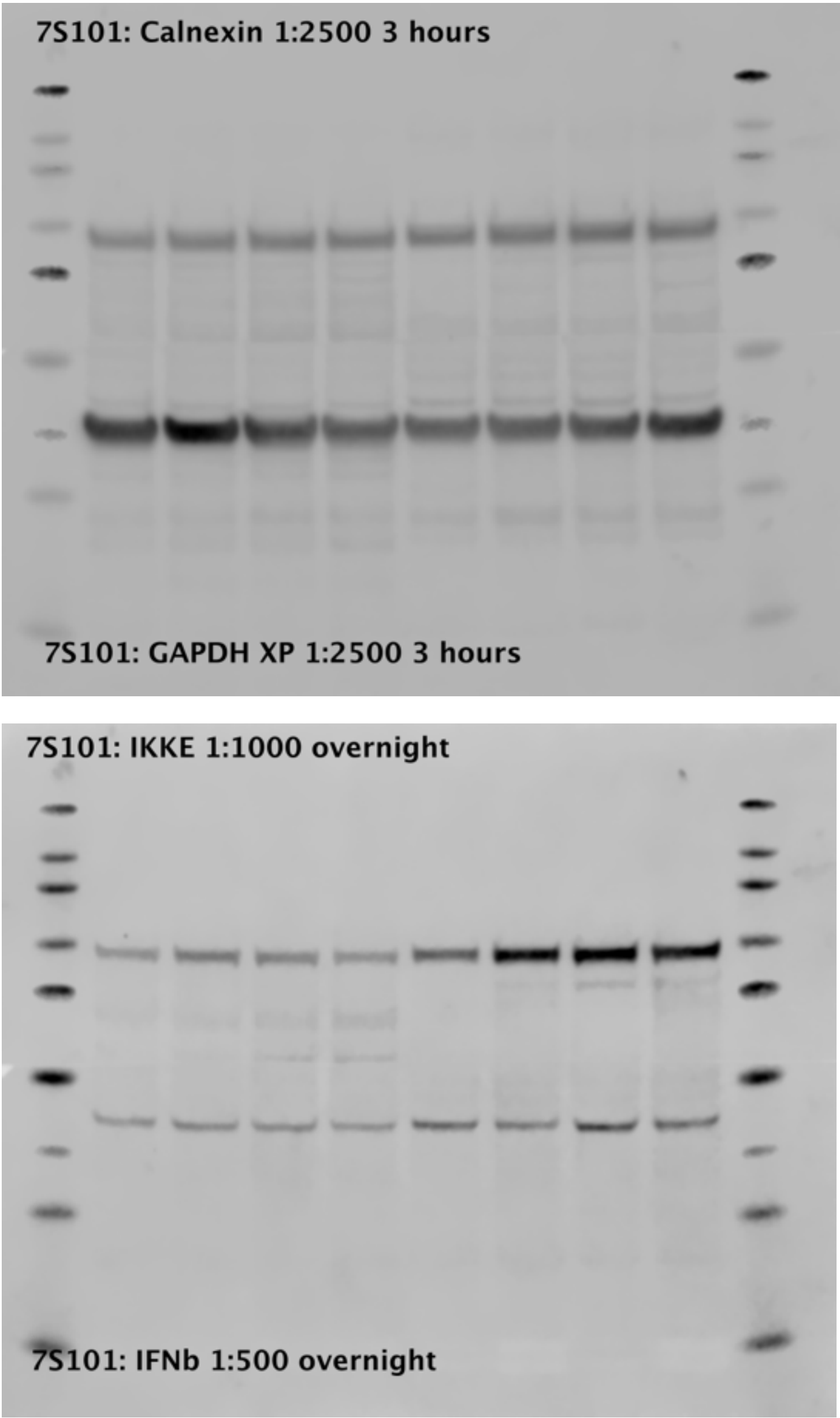

Fig 2A

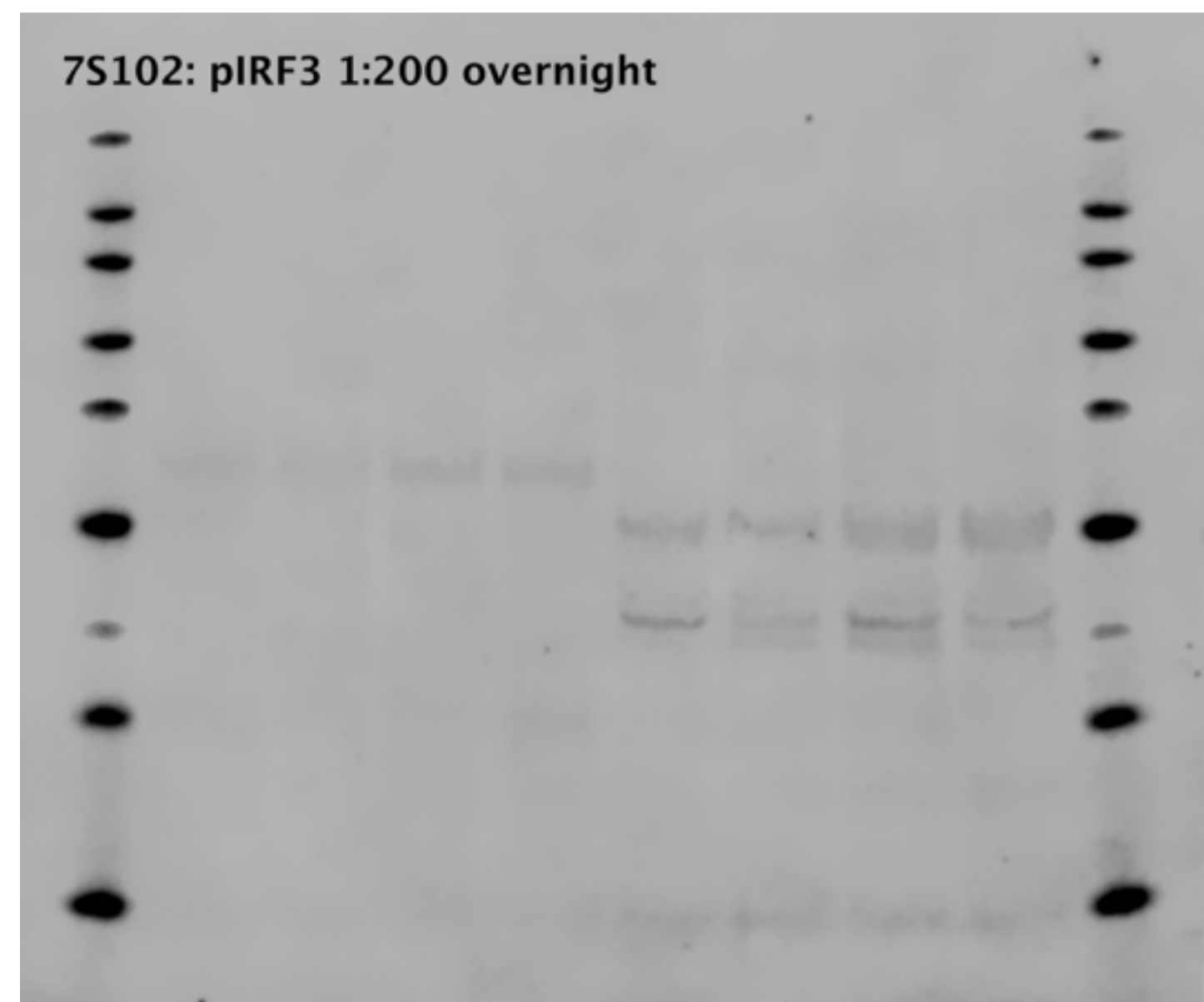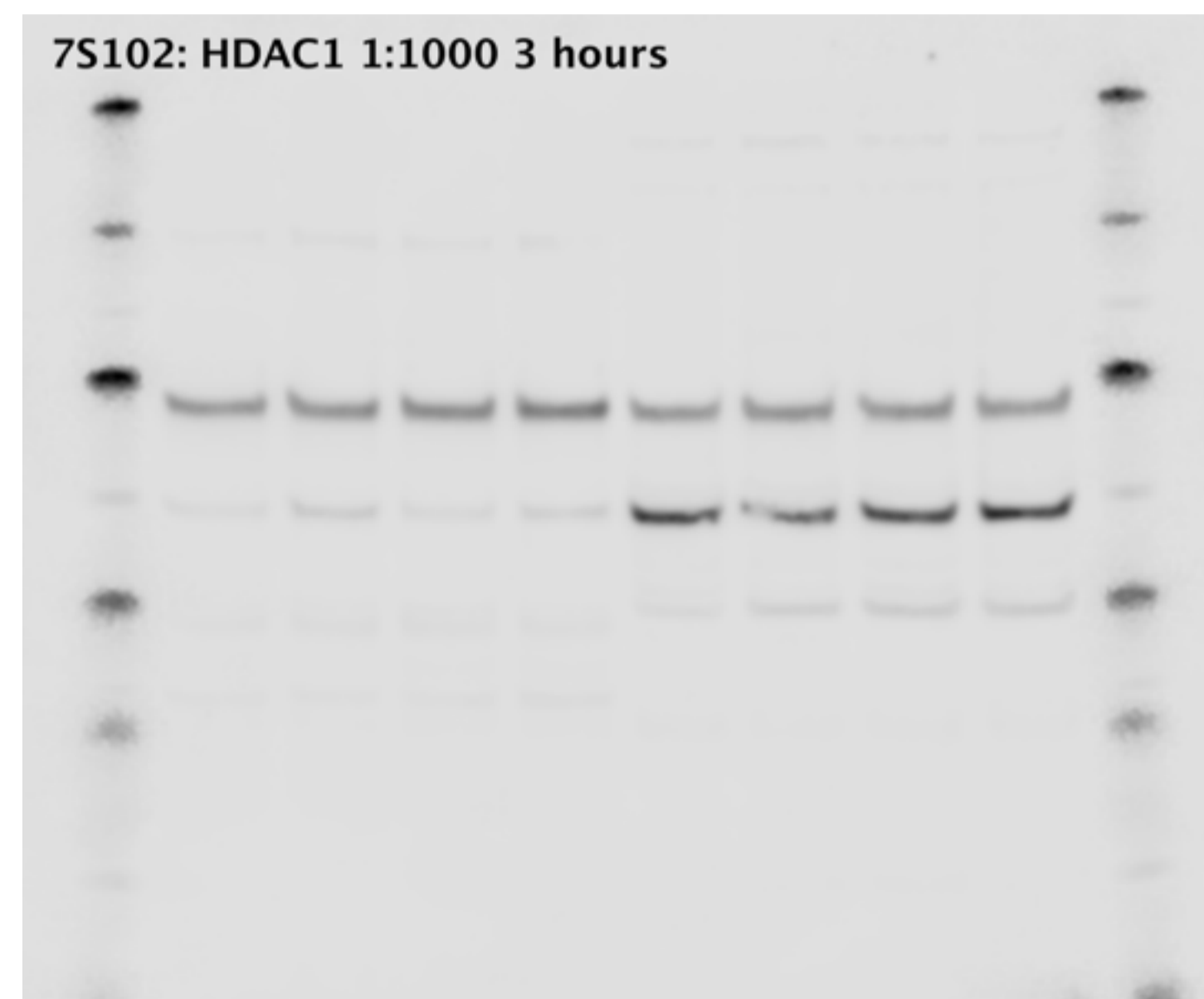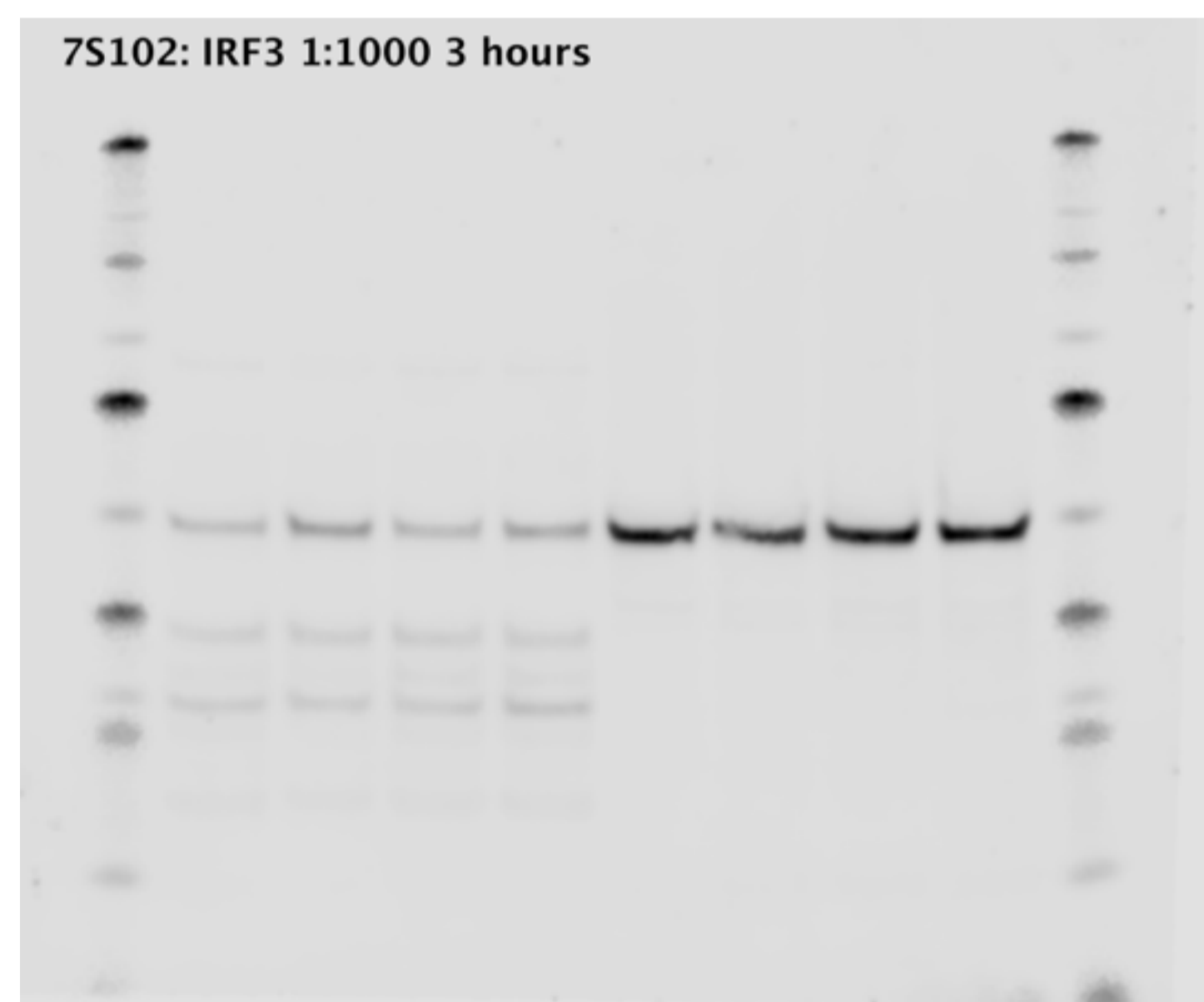

Fig 2B

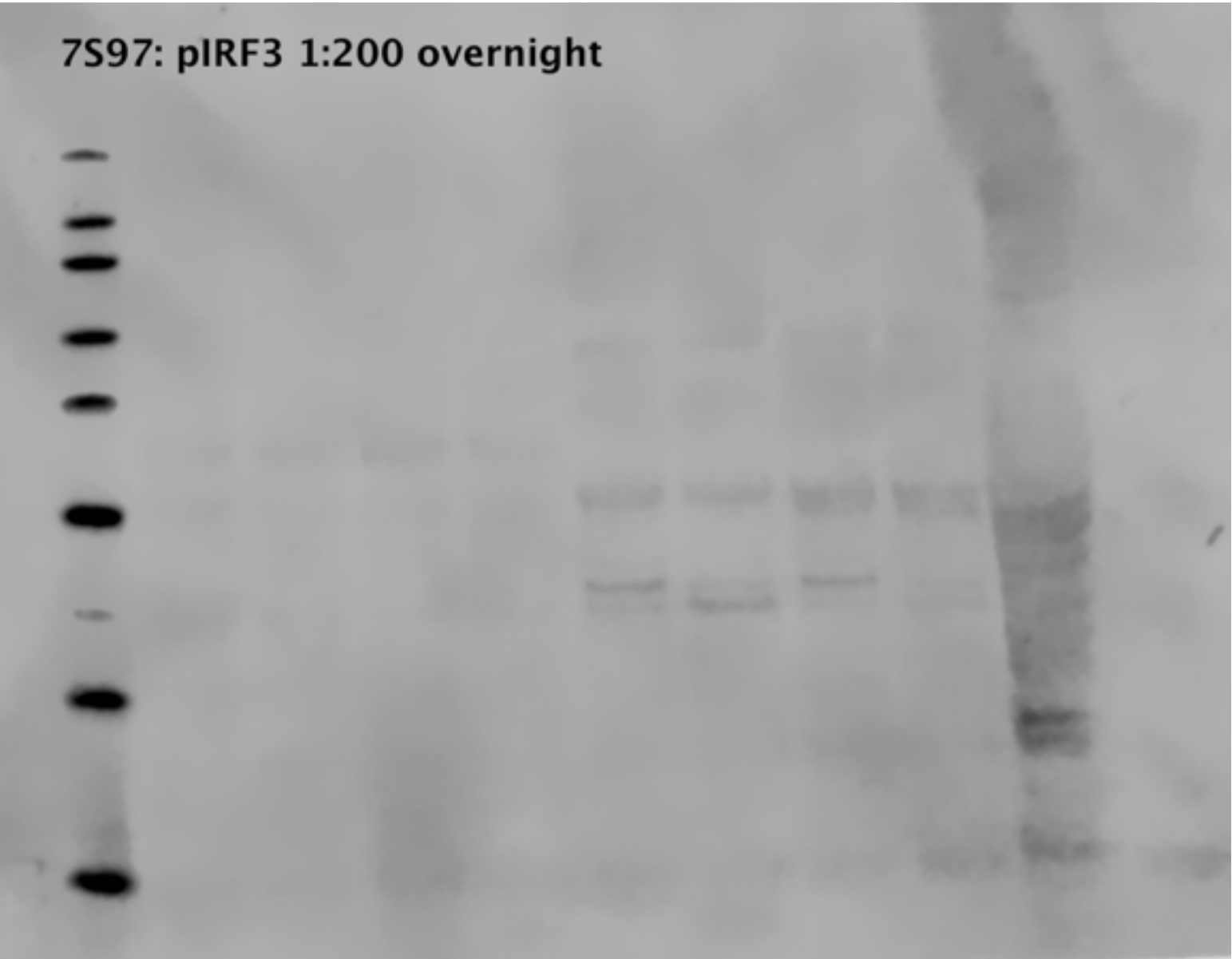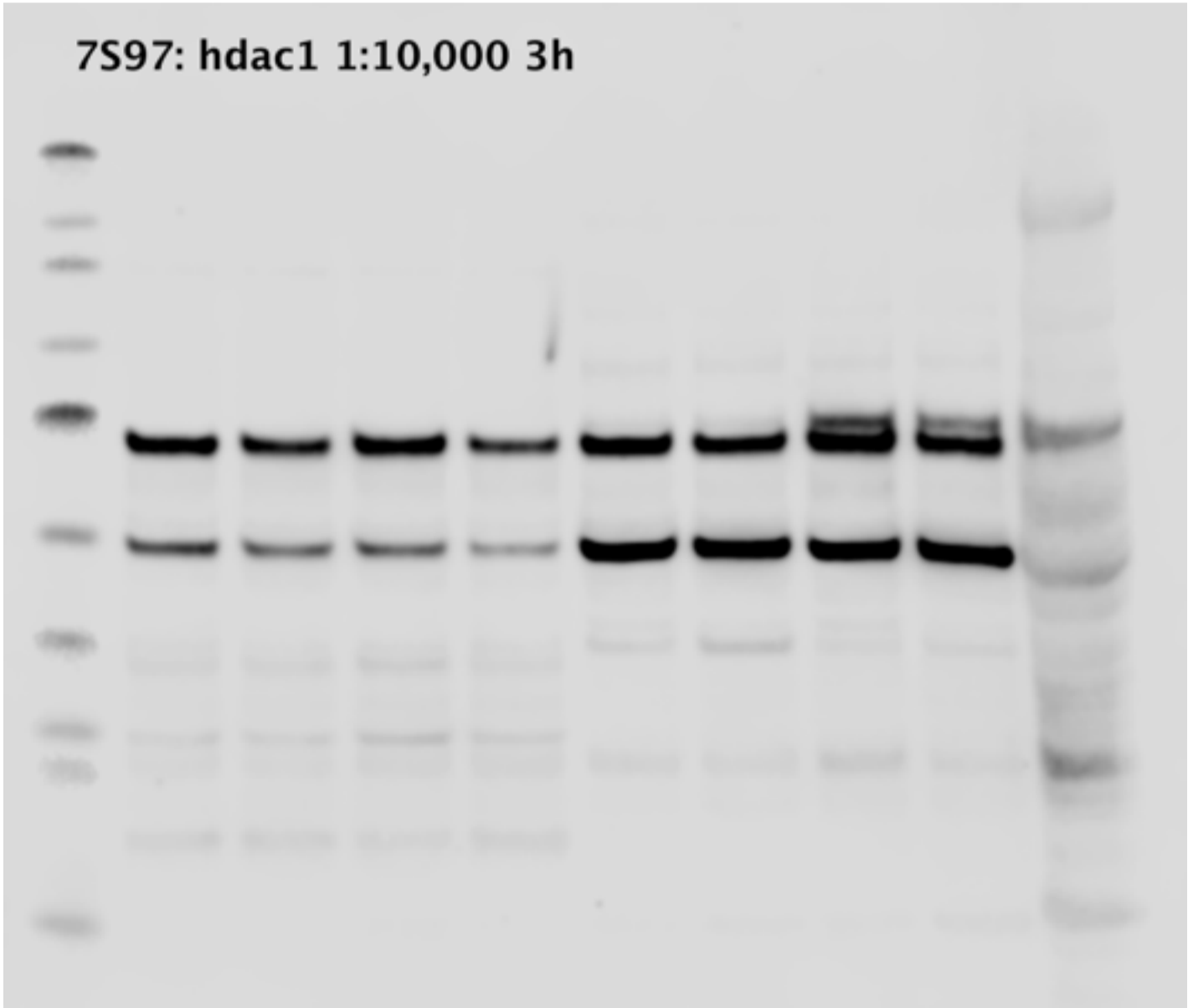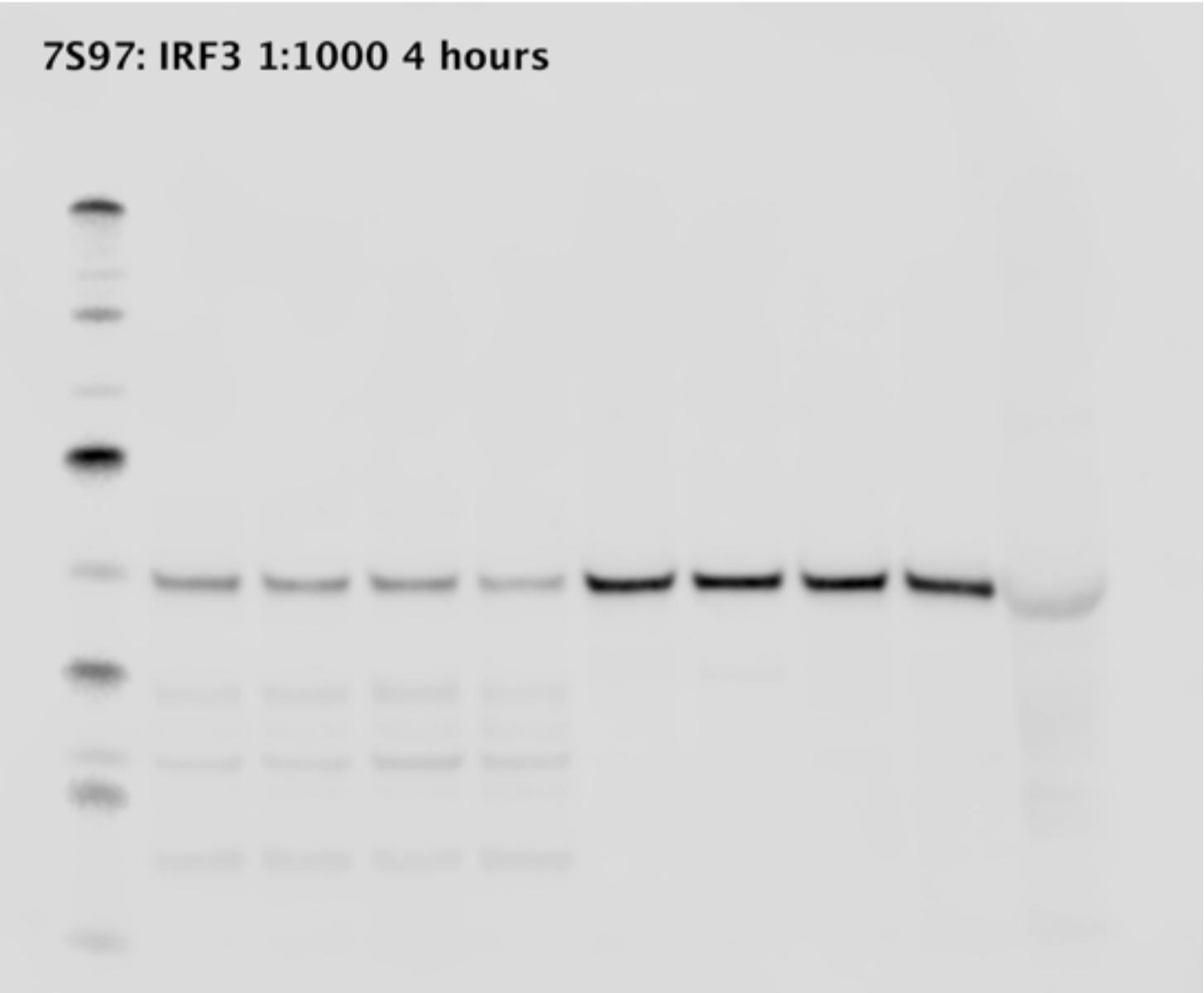

Fig 2F

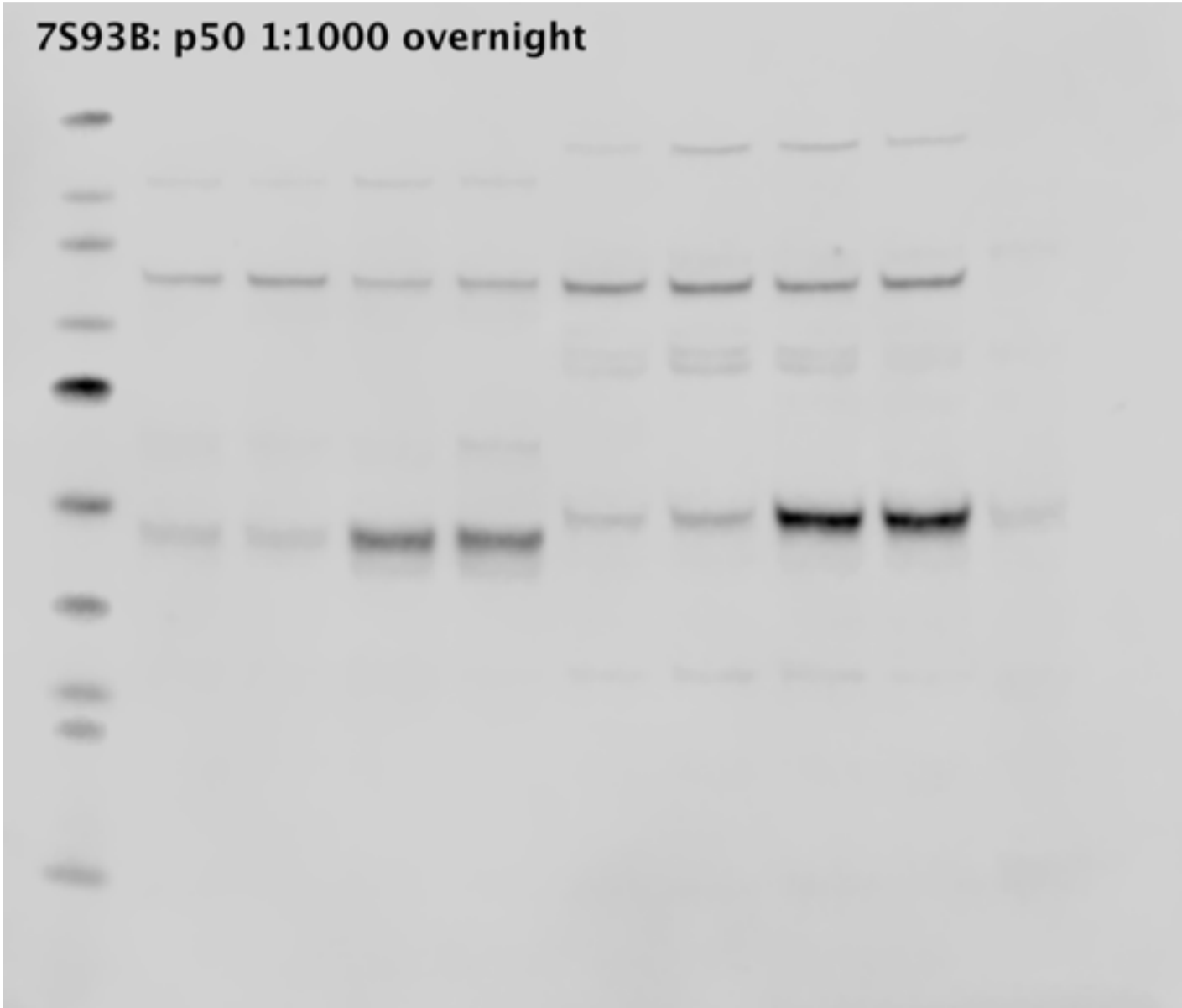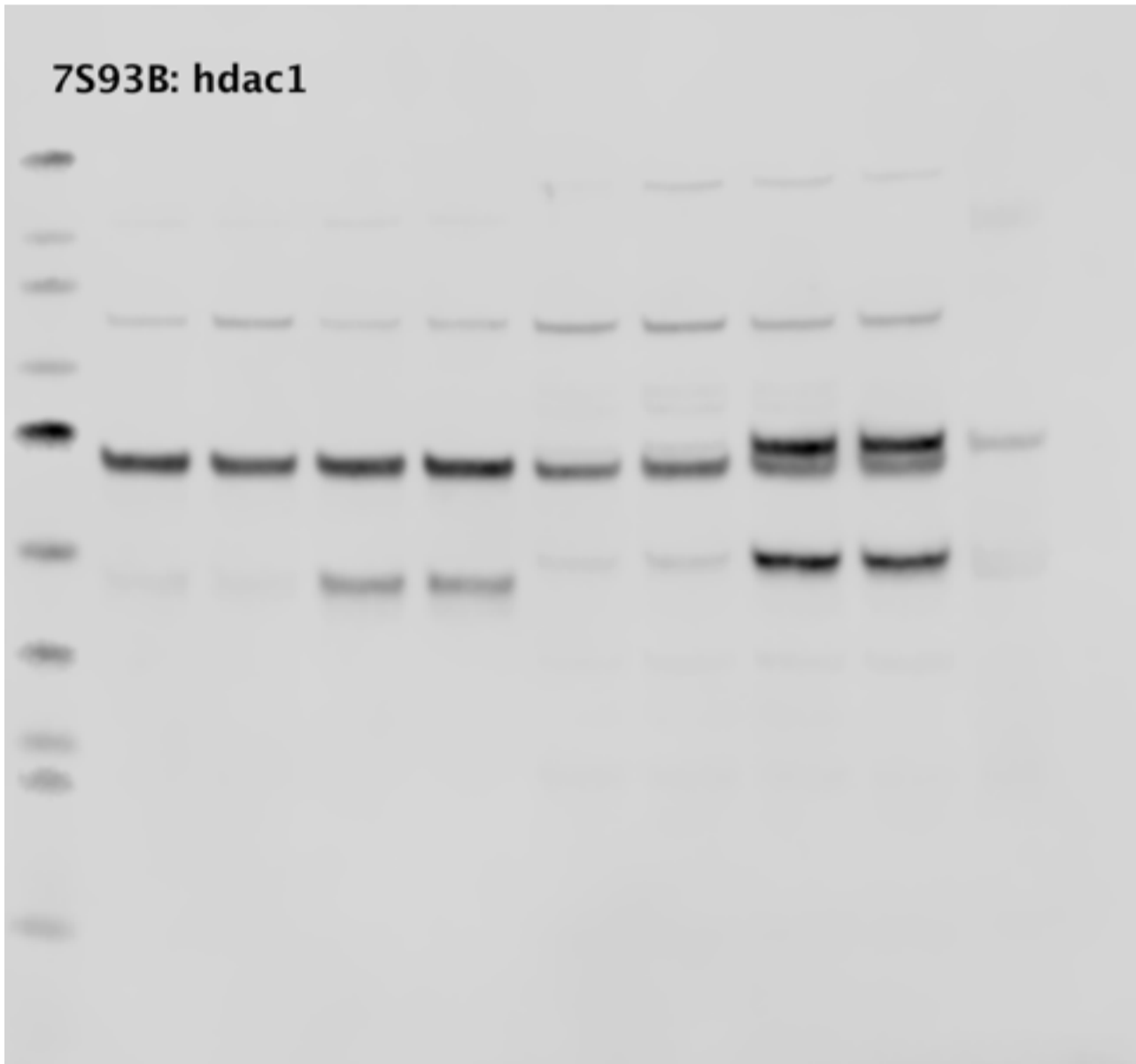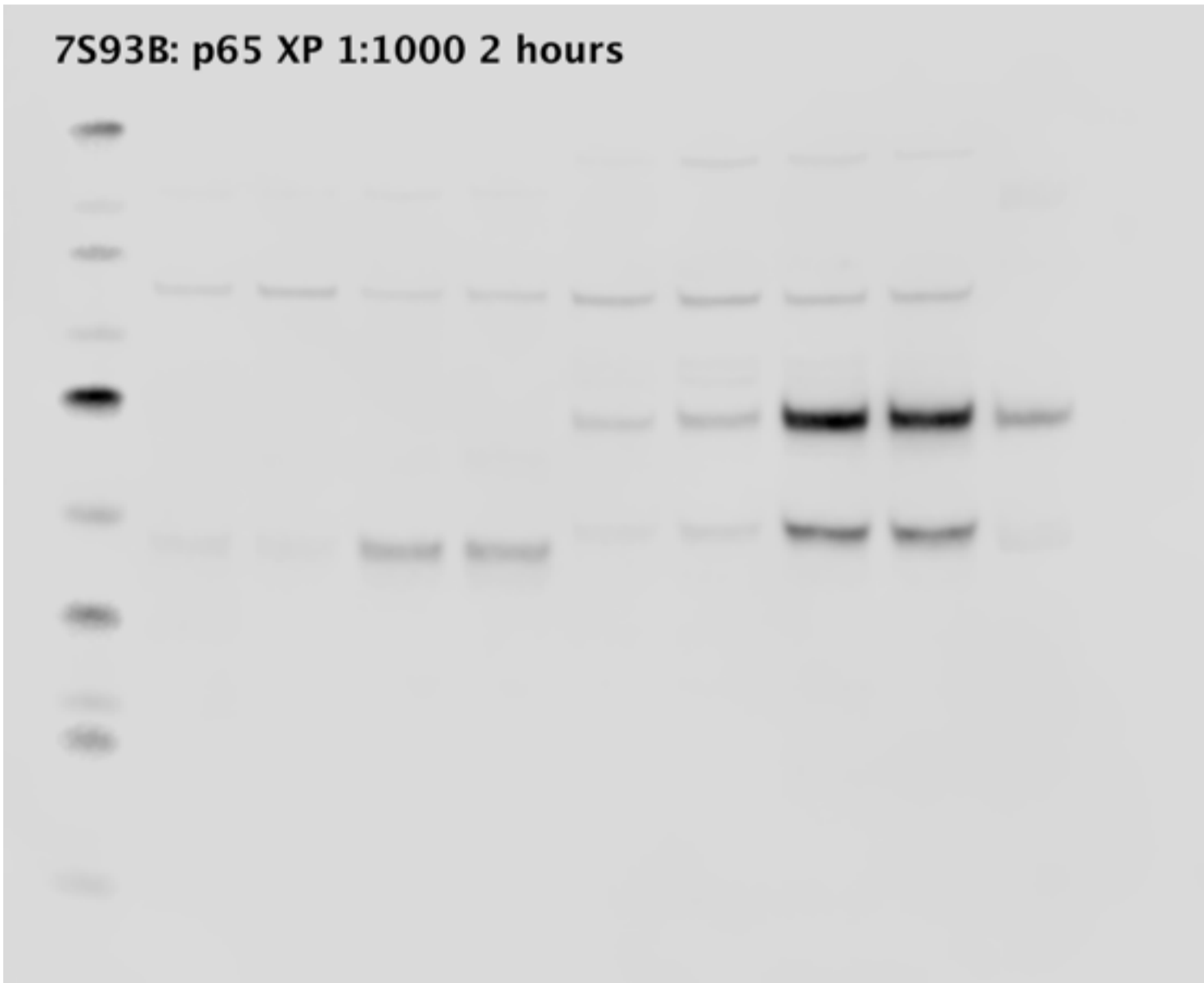

Fig 2G

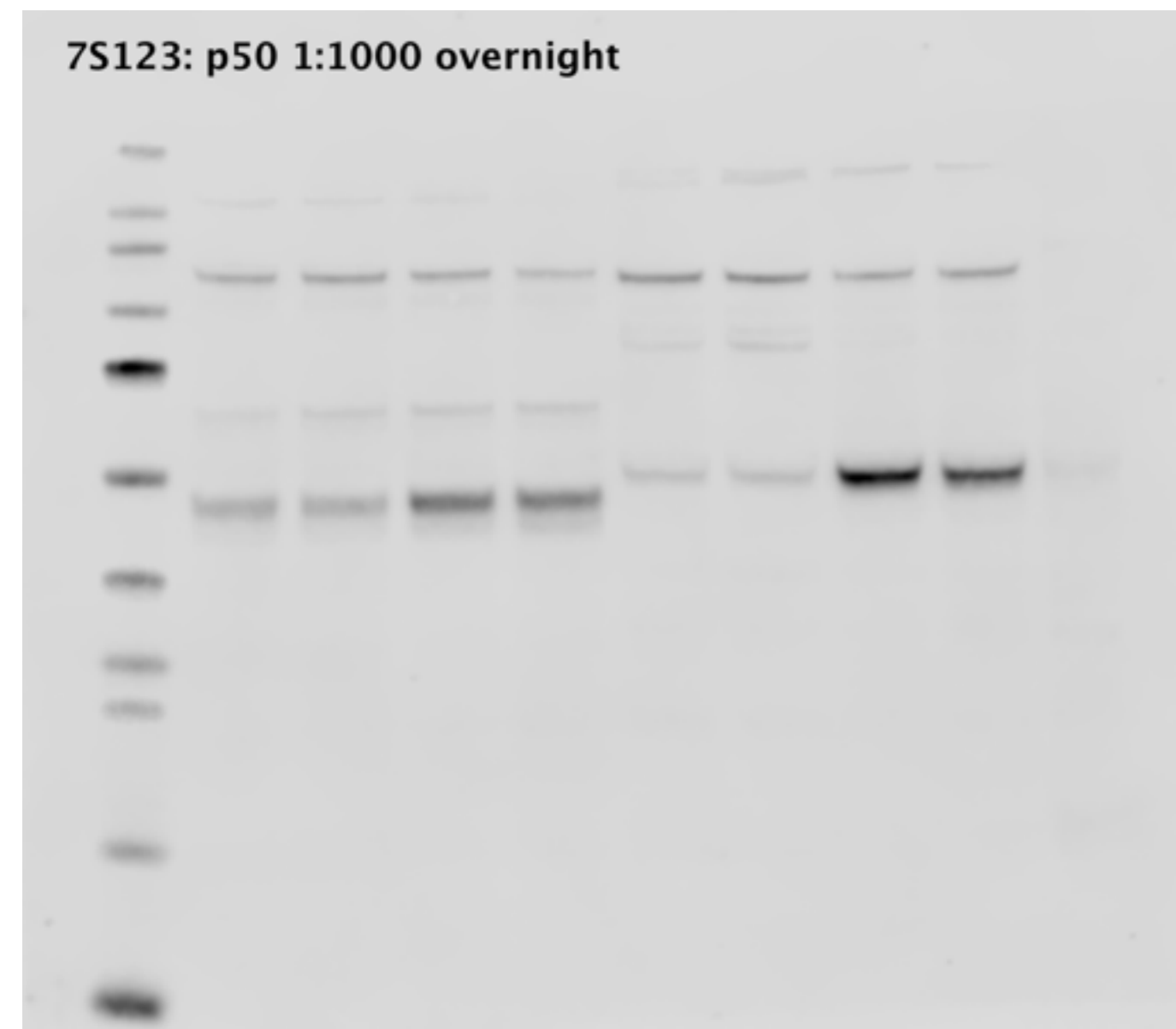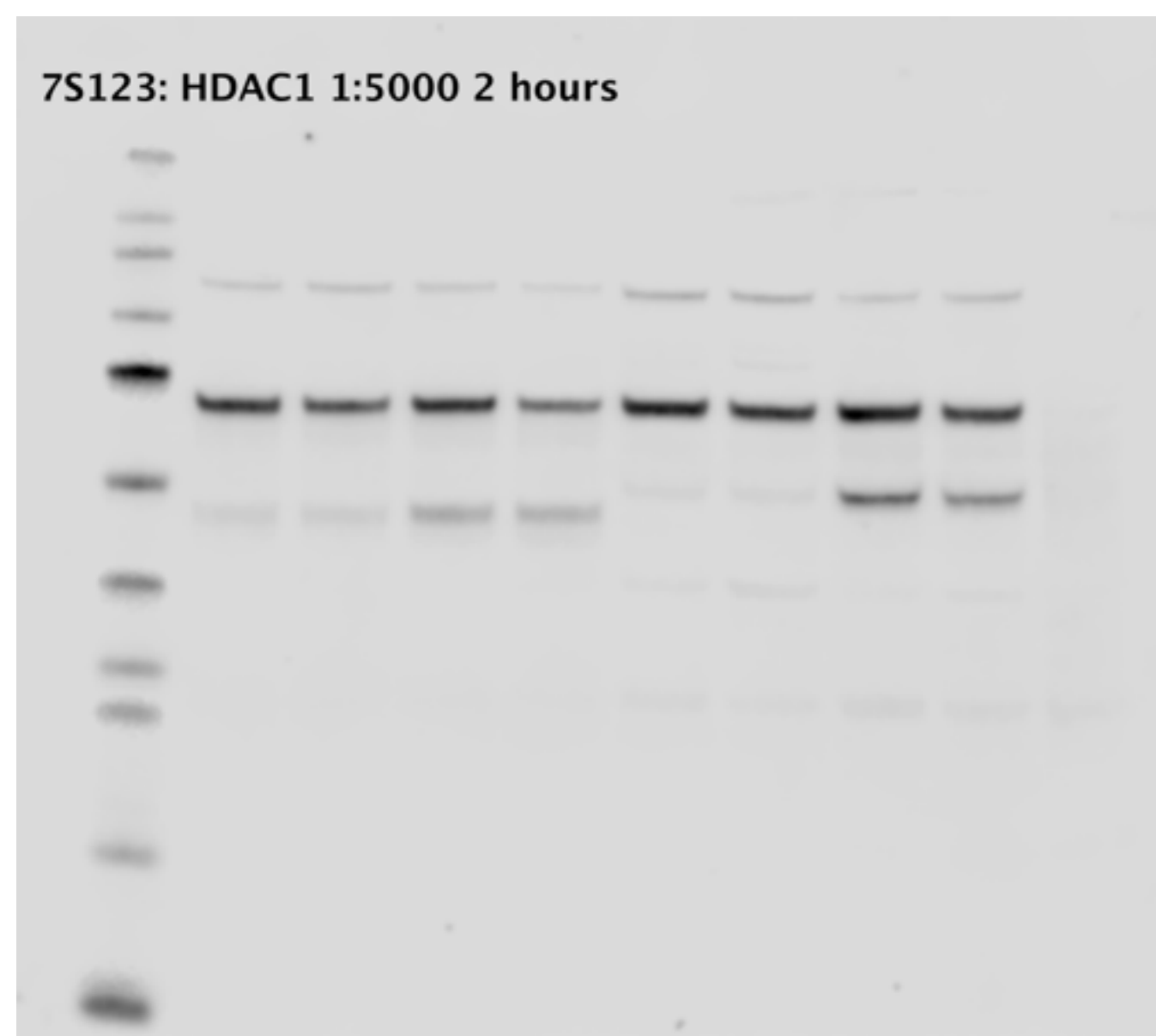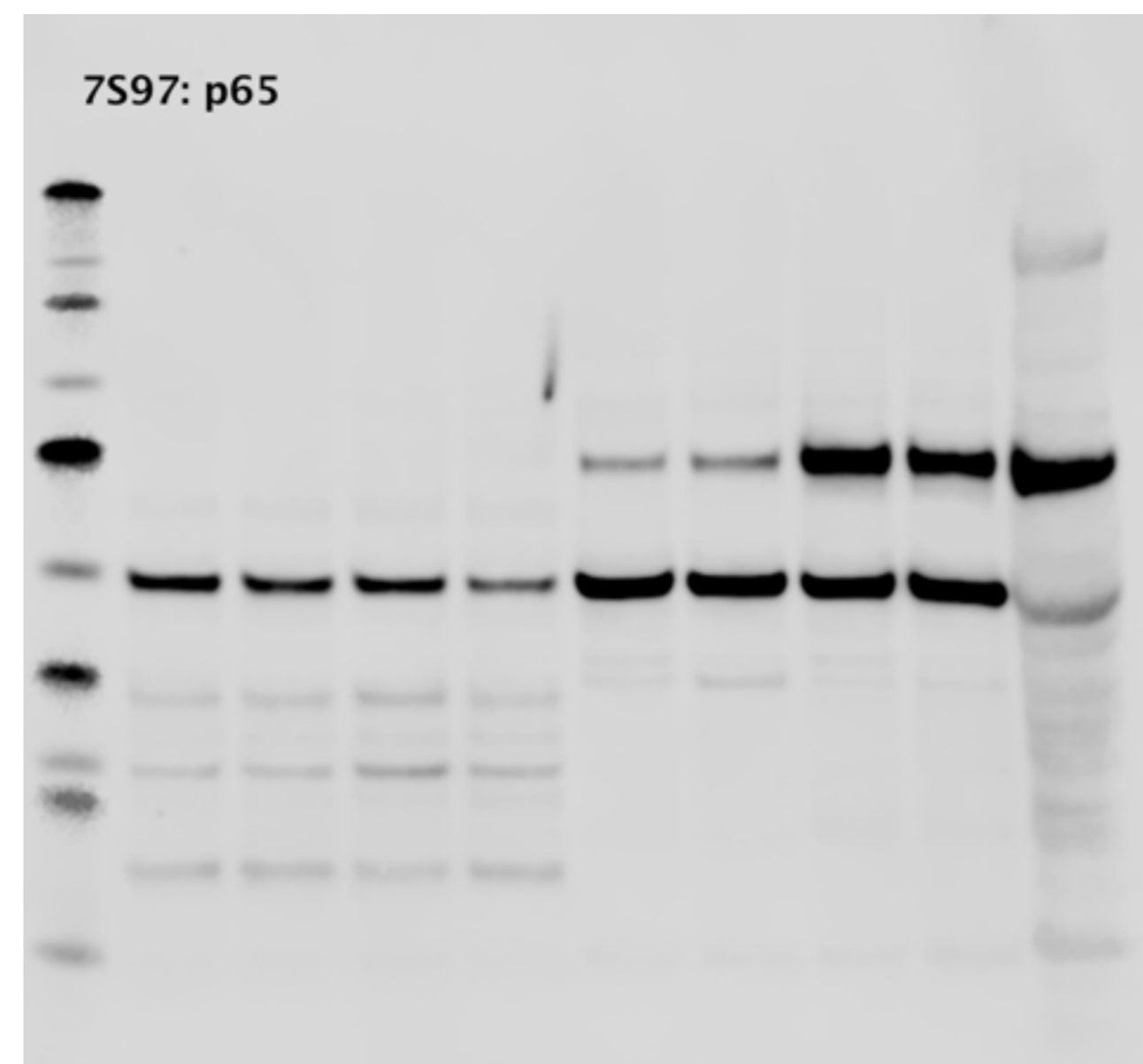

Fig 2K

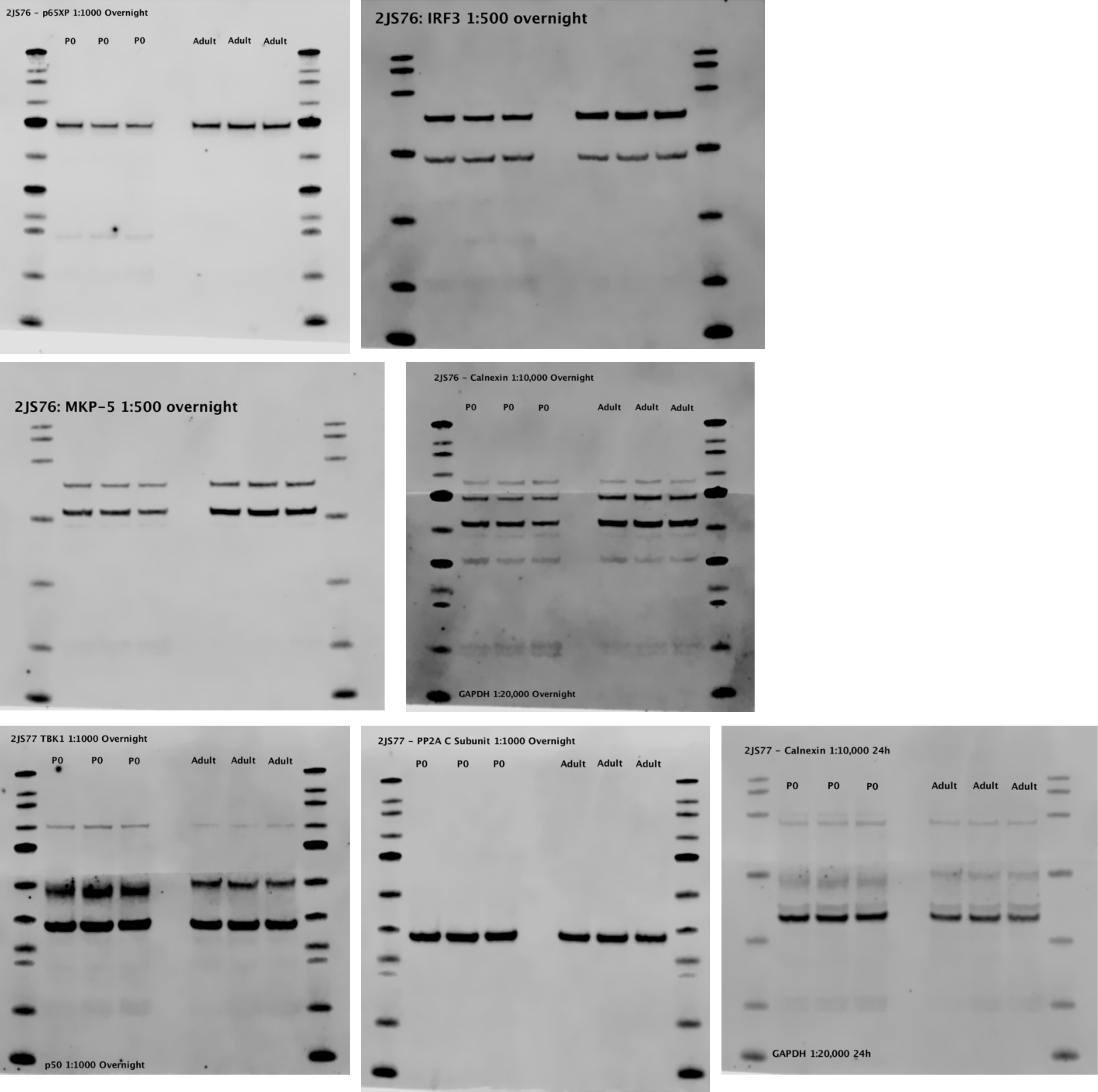

Fig 4A

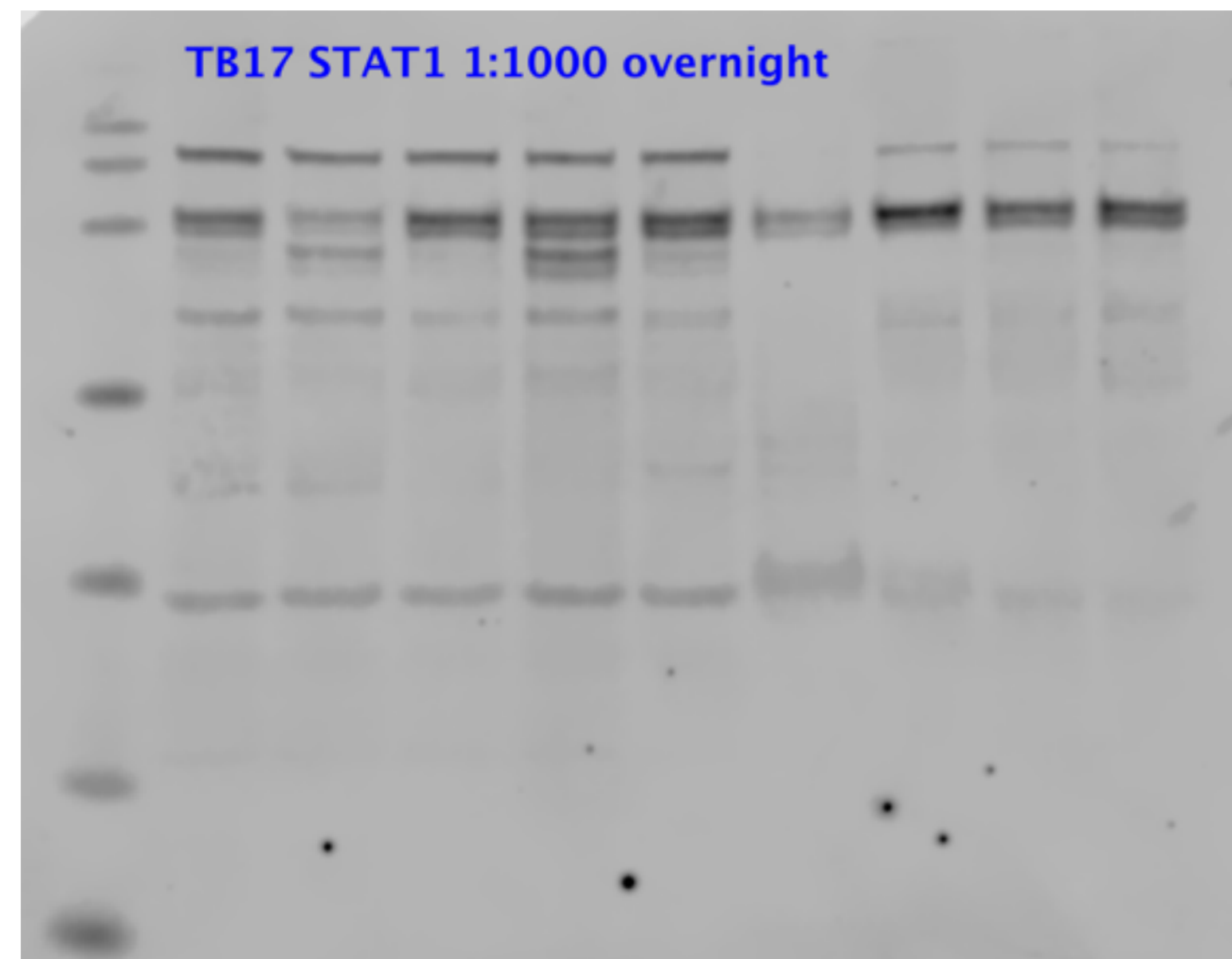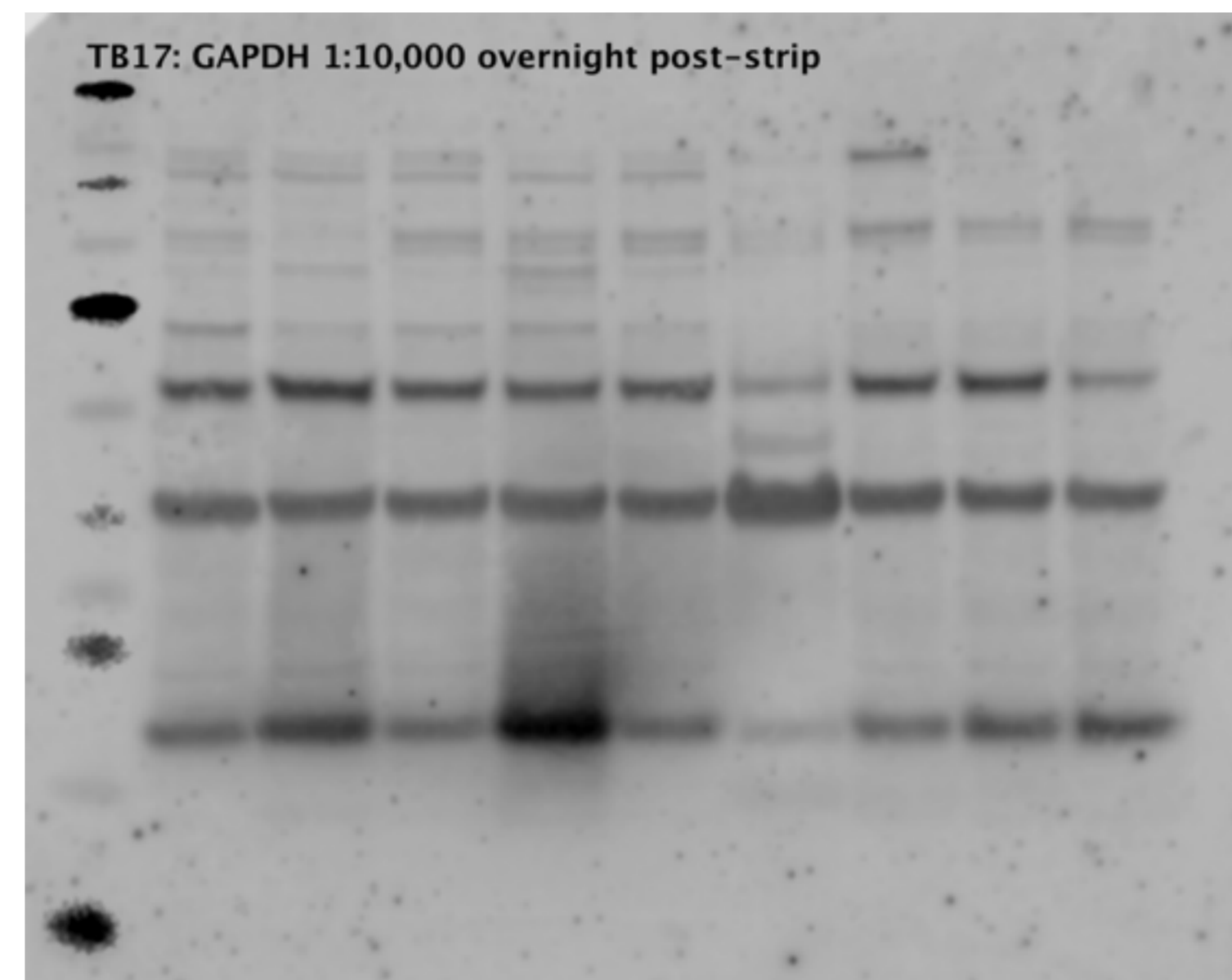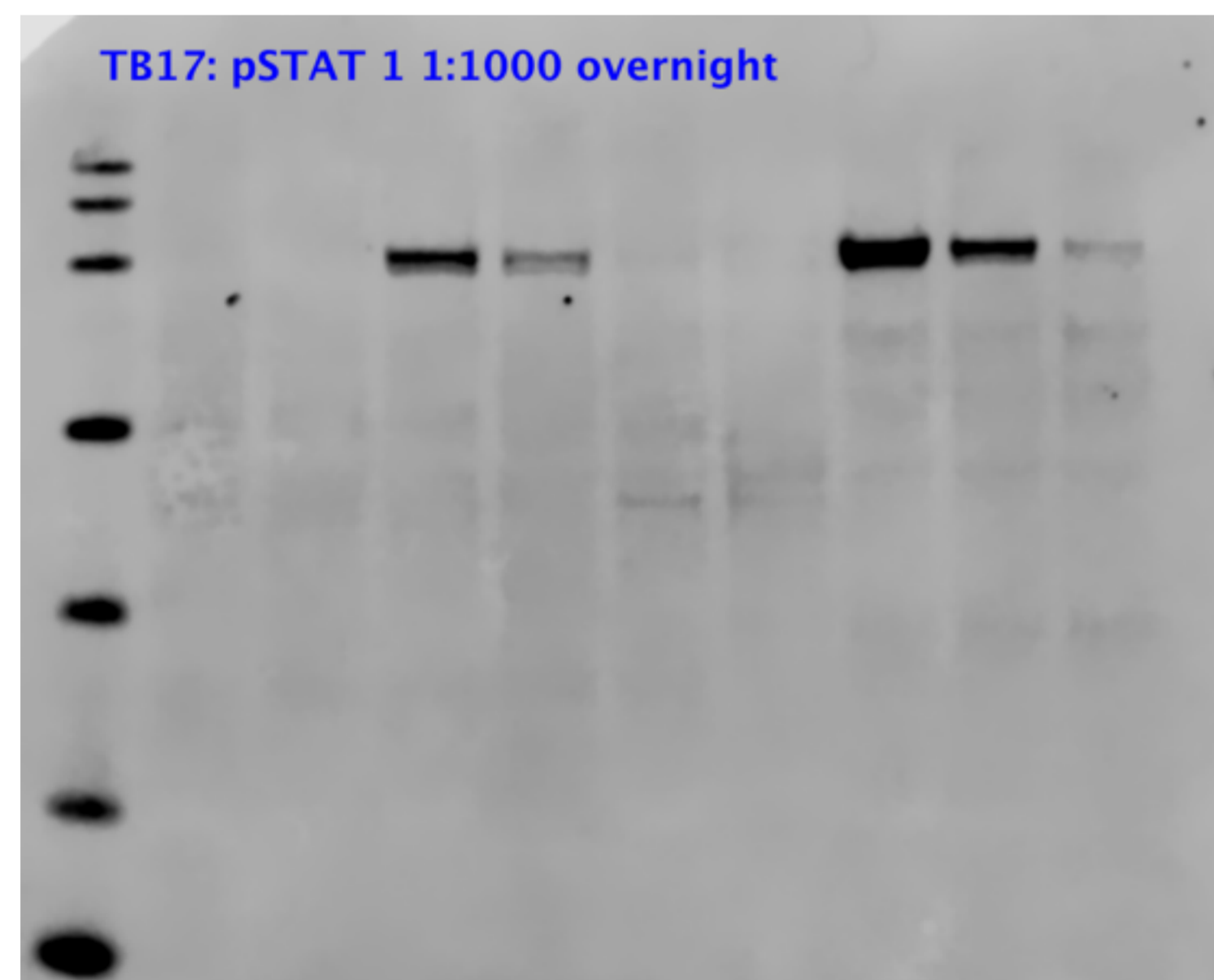

Fig 4B

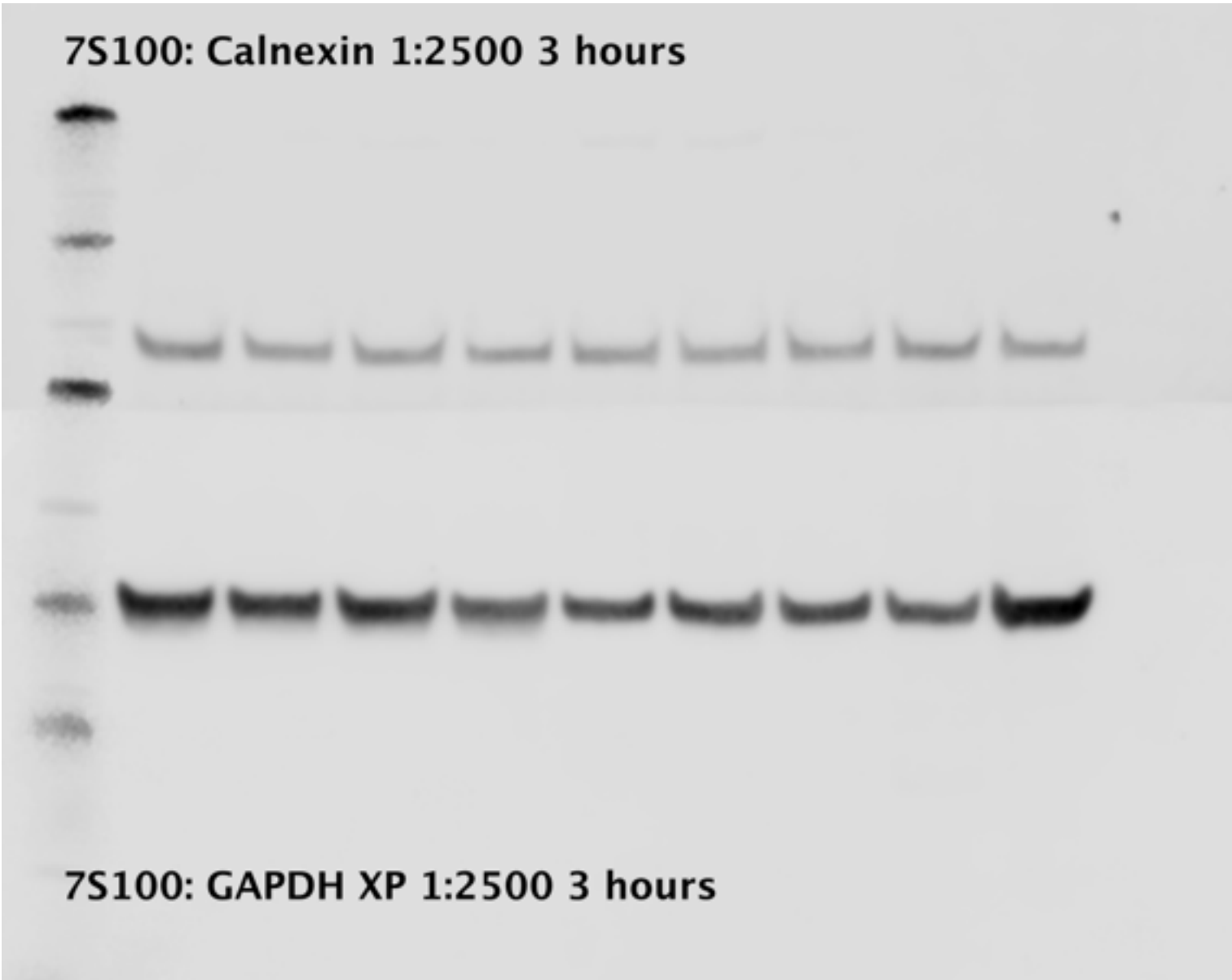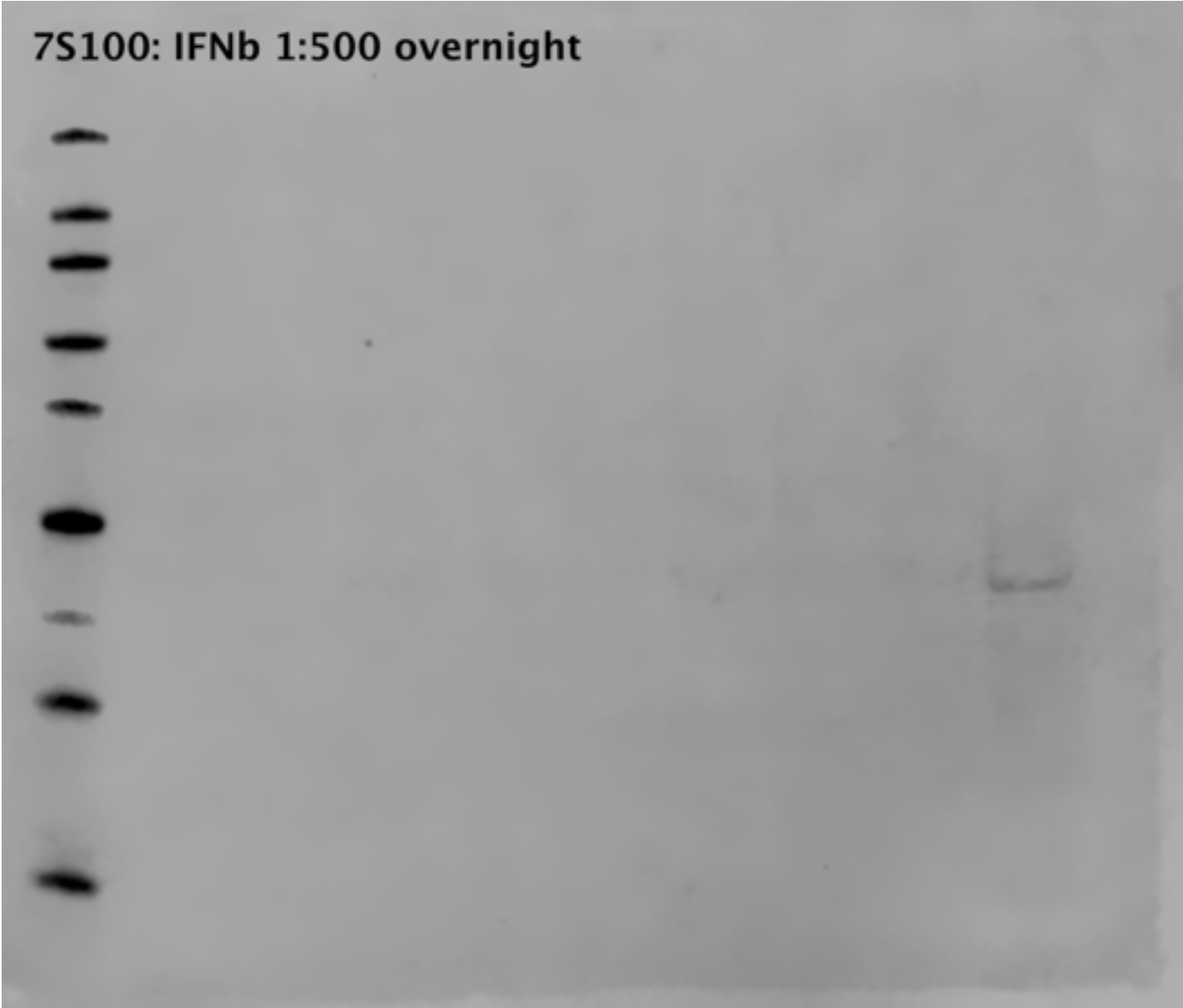

Fig 5A

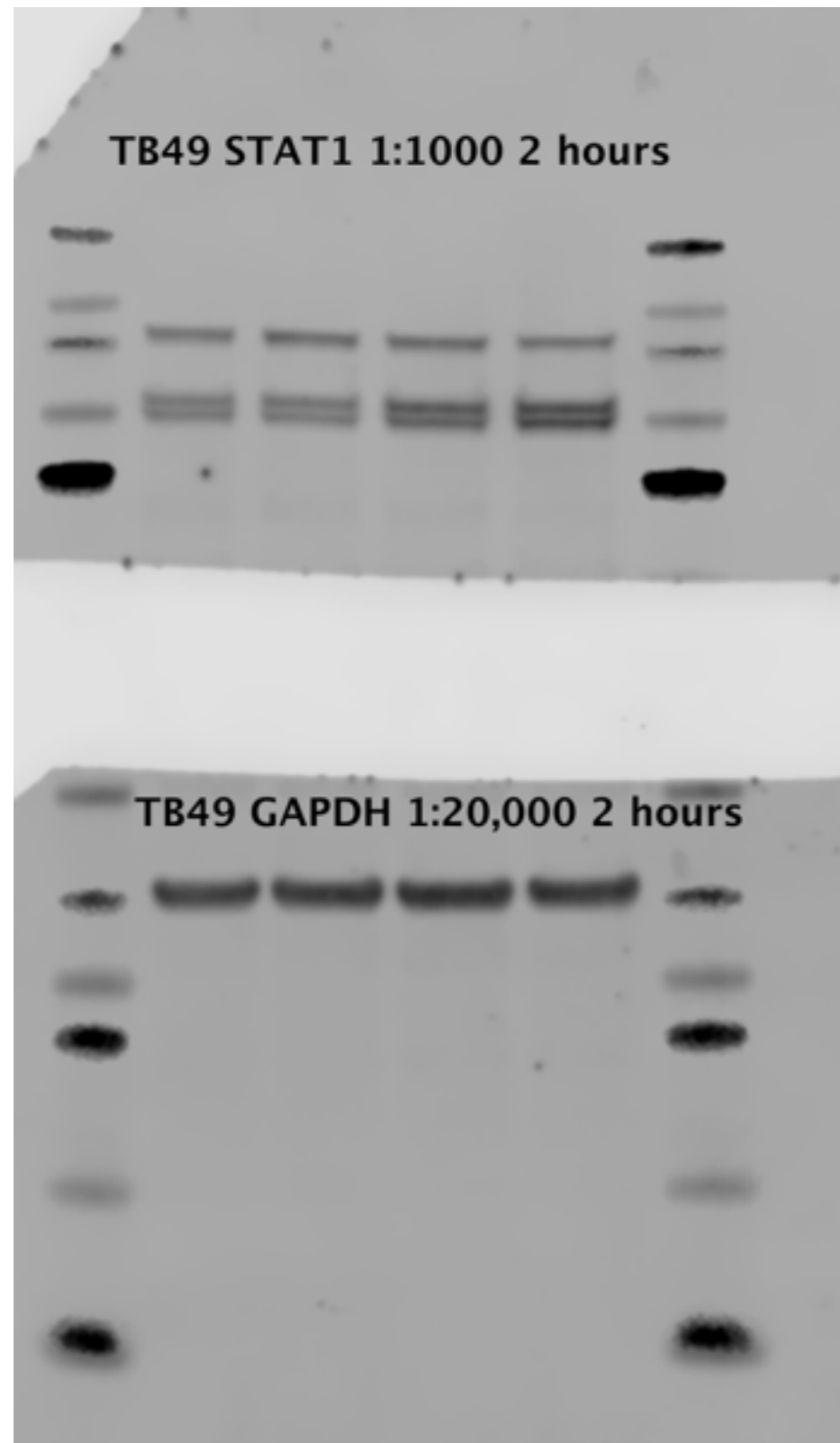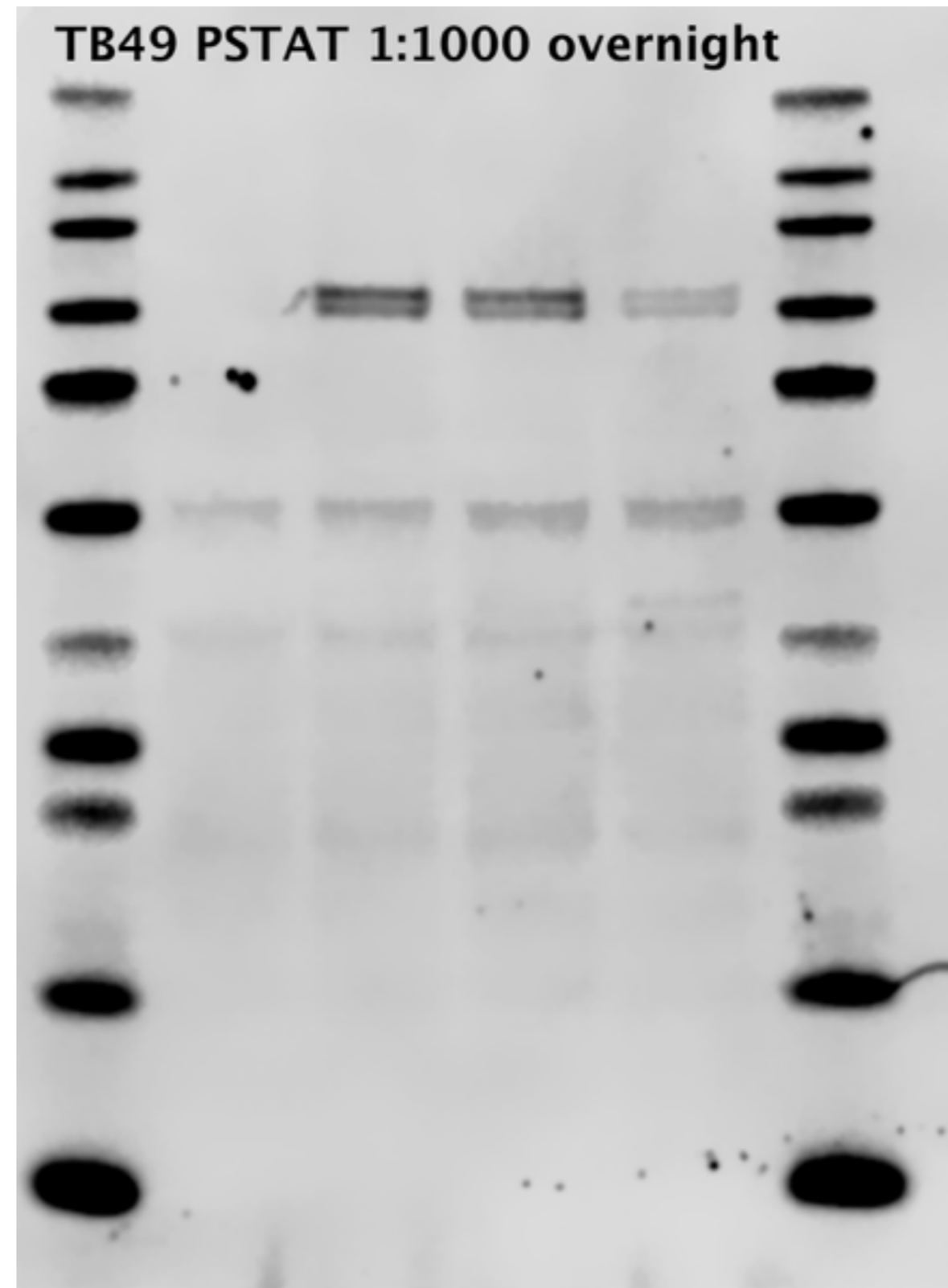

Fig 5B

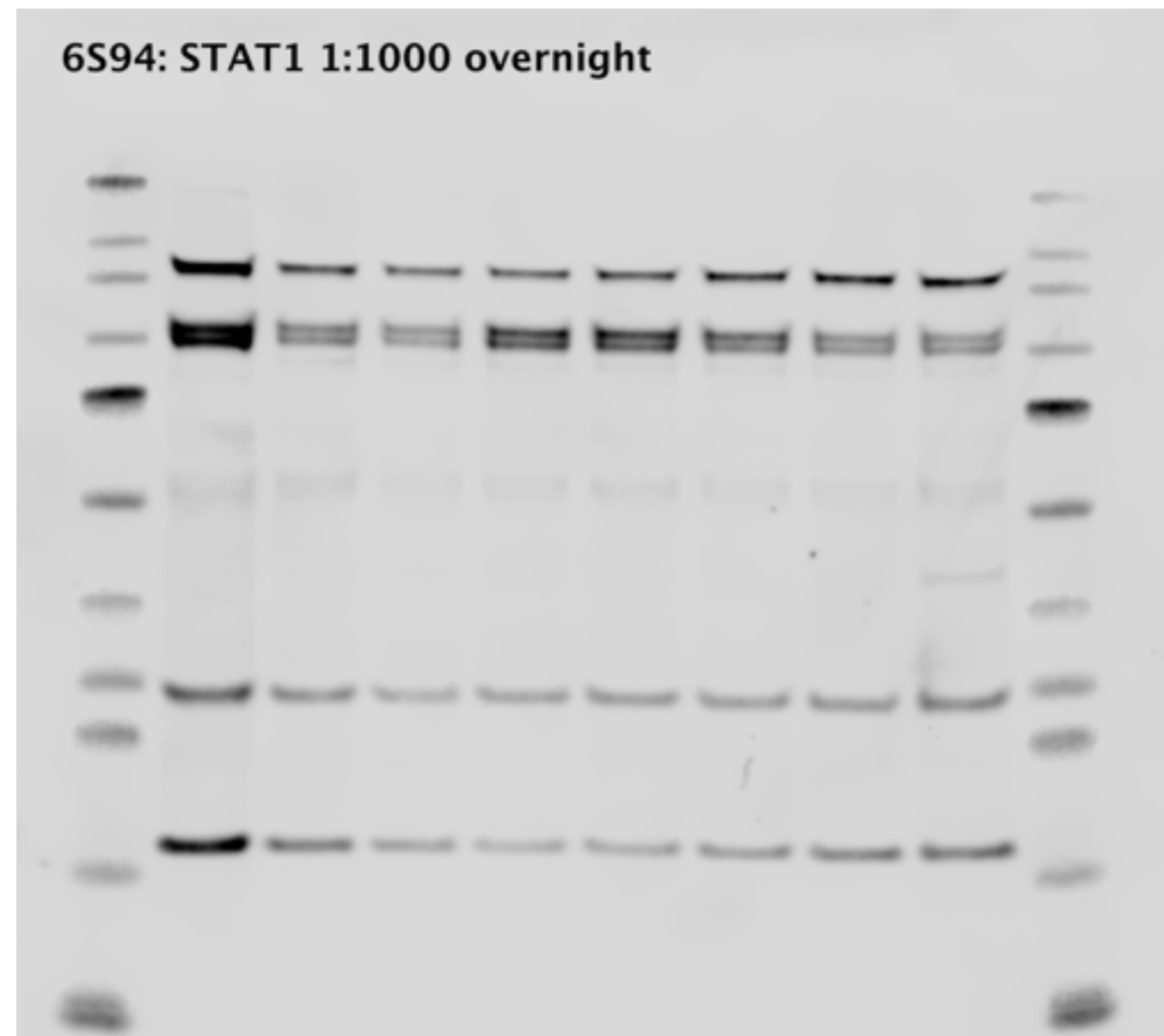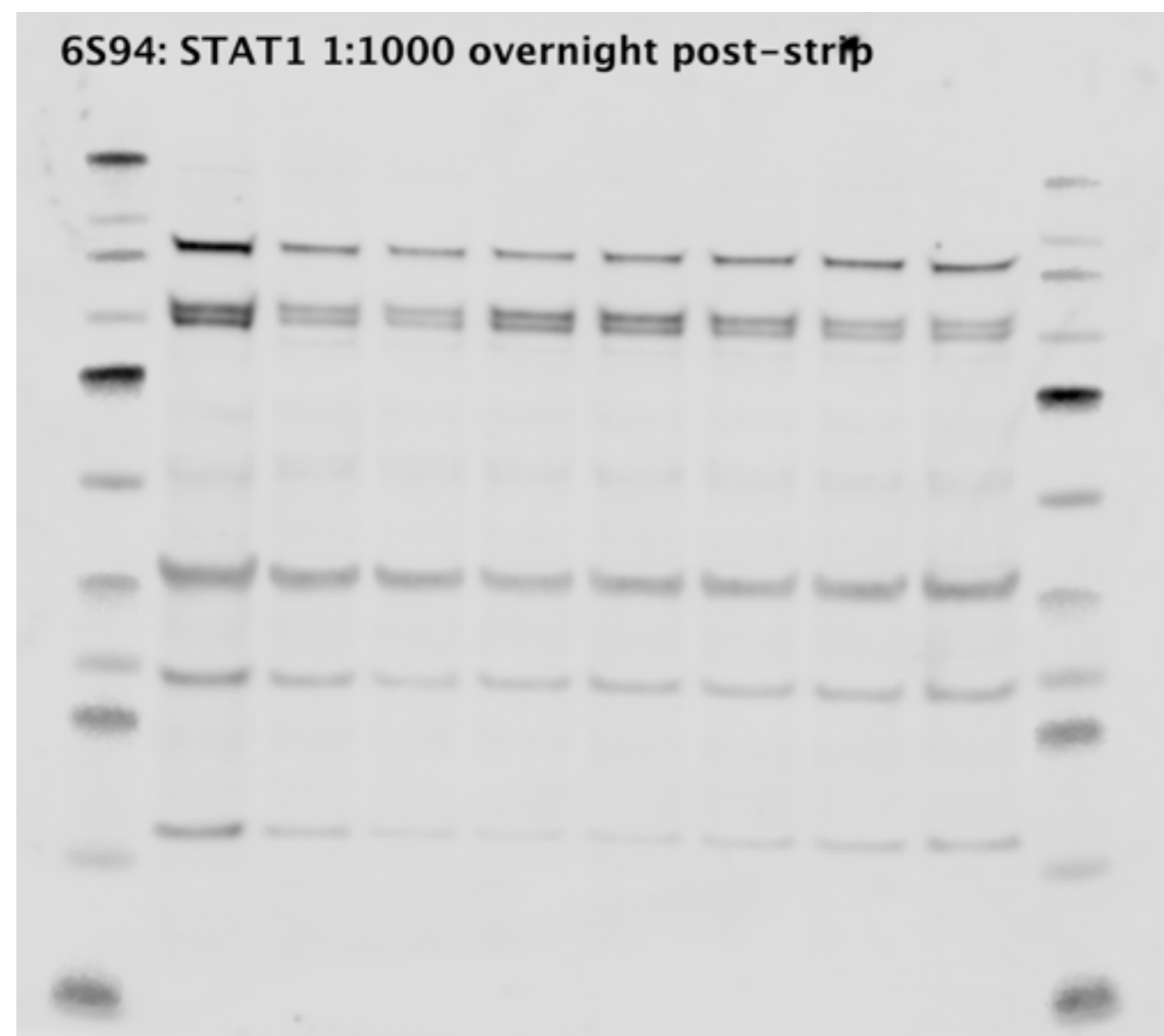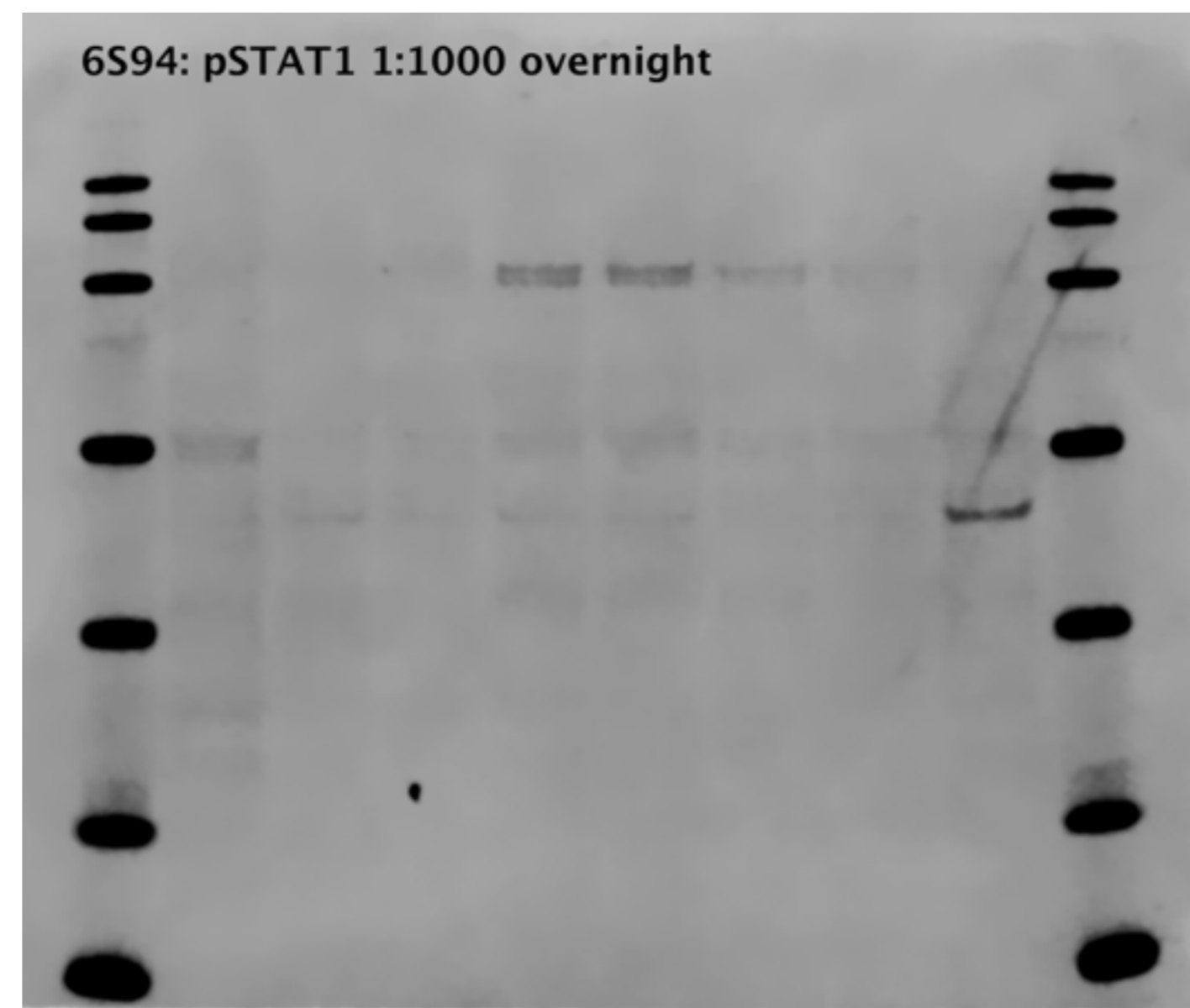

Fig 5C

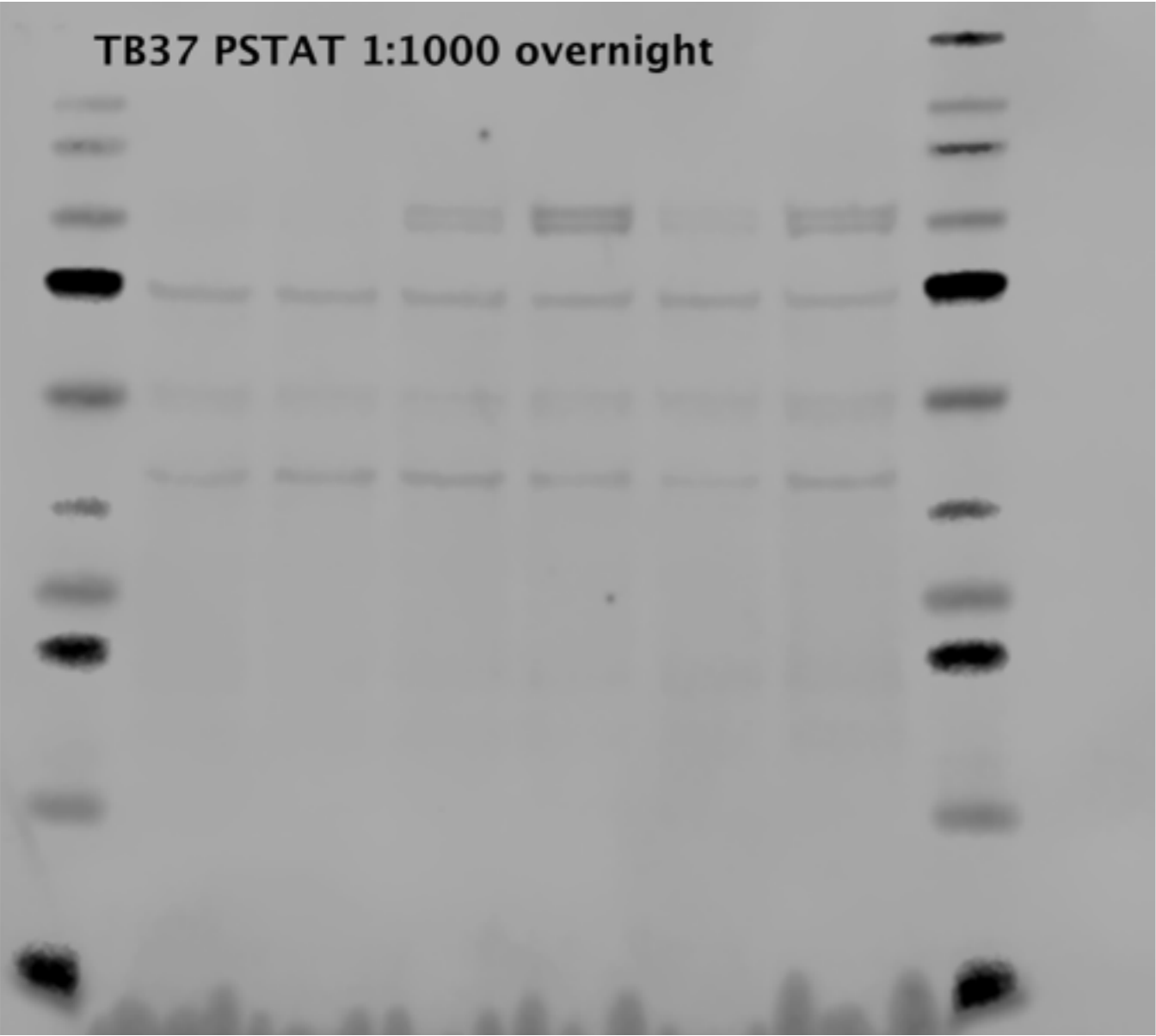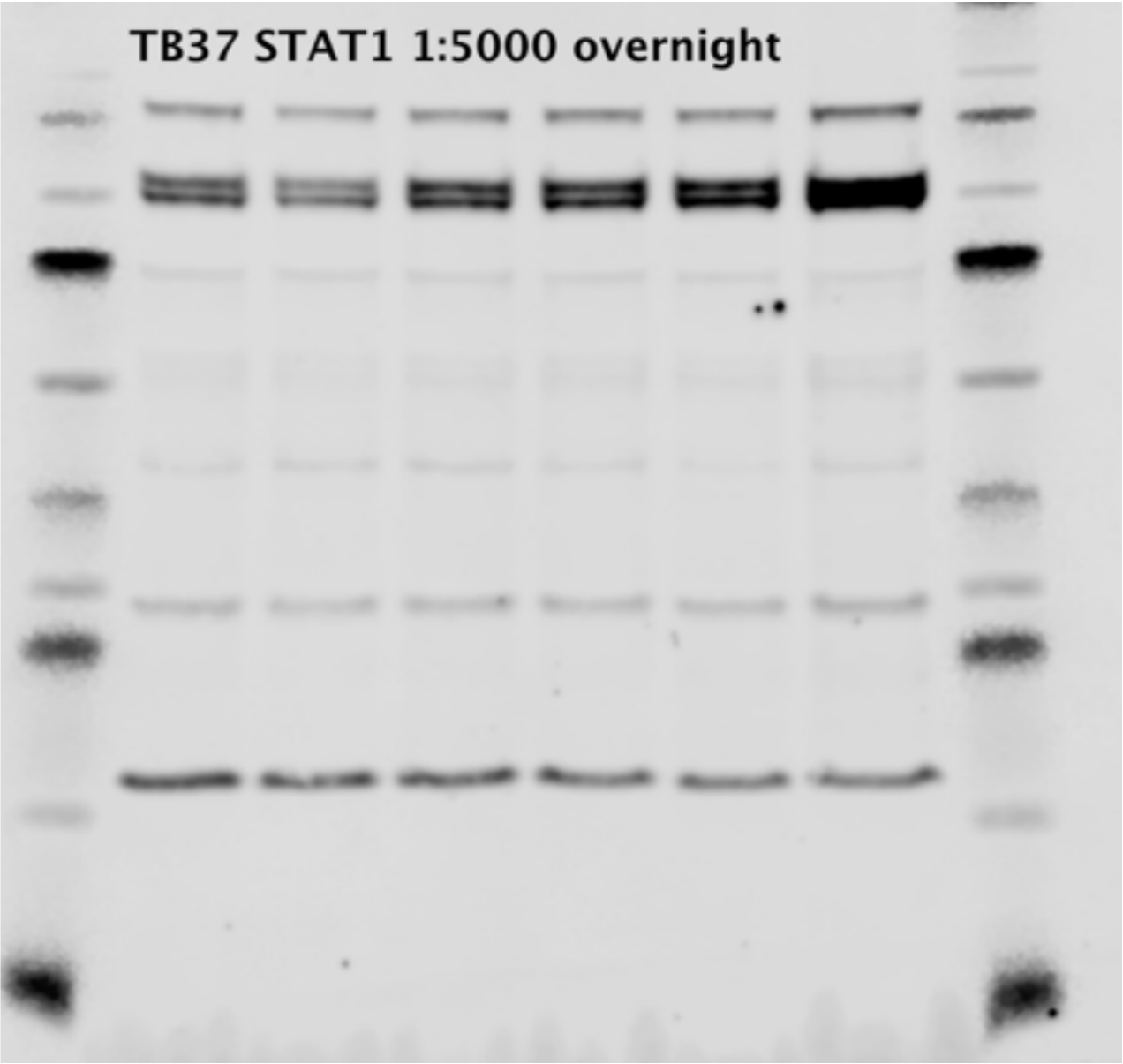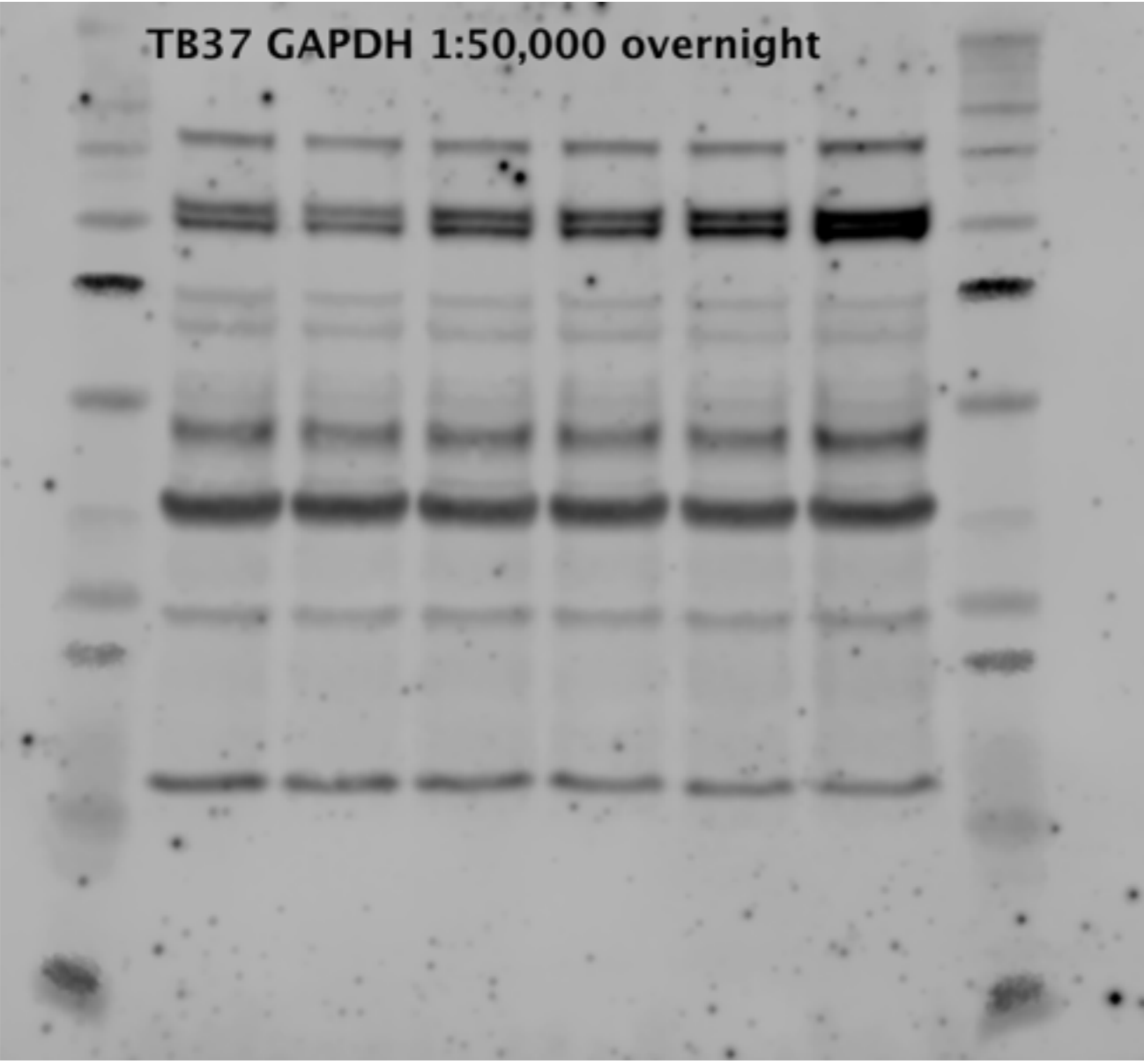

Fig 5E

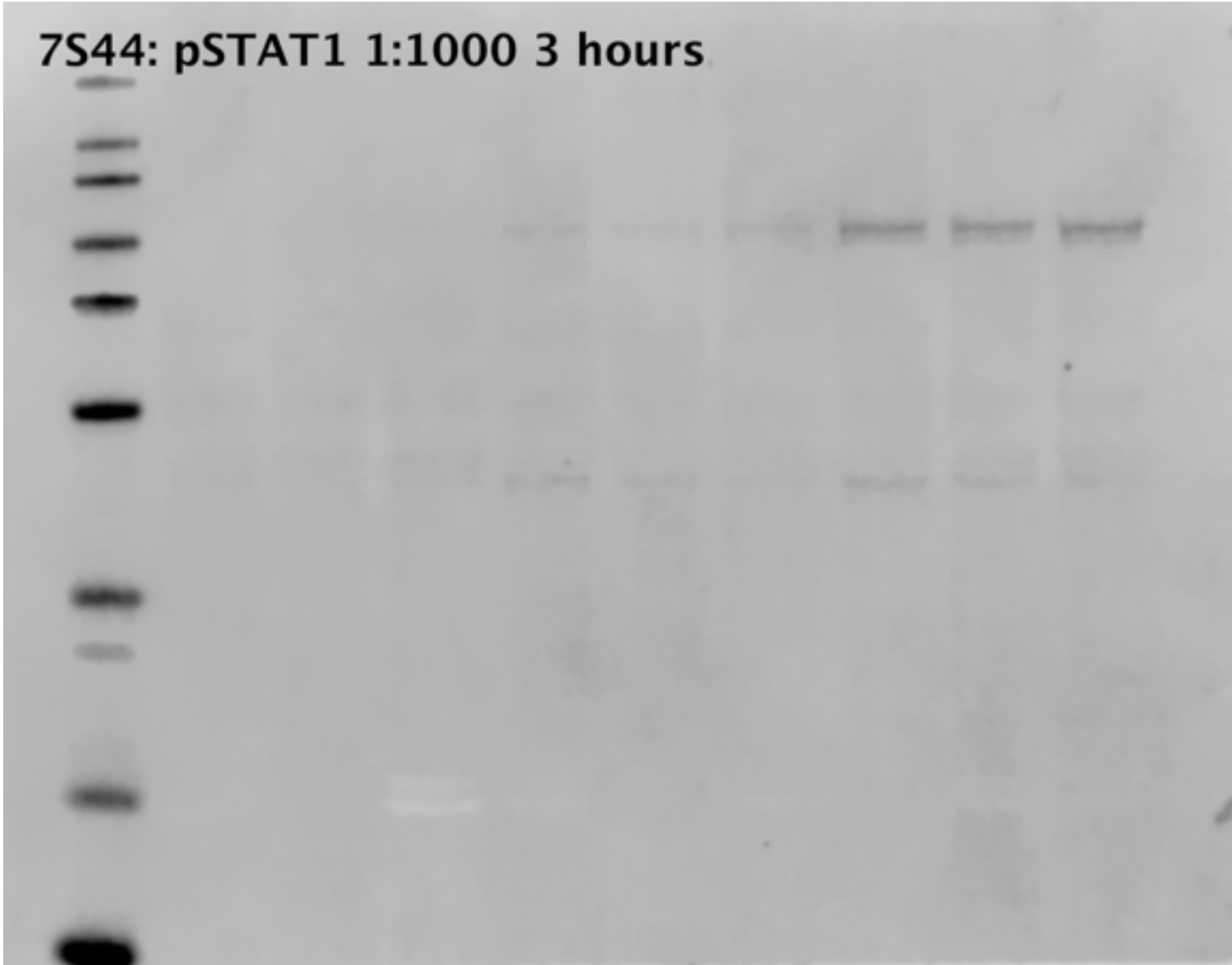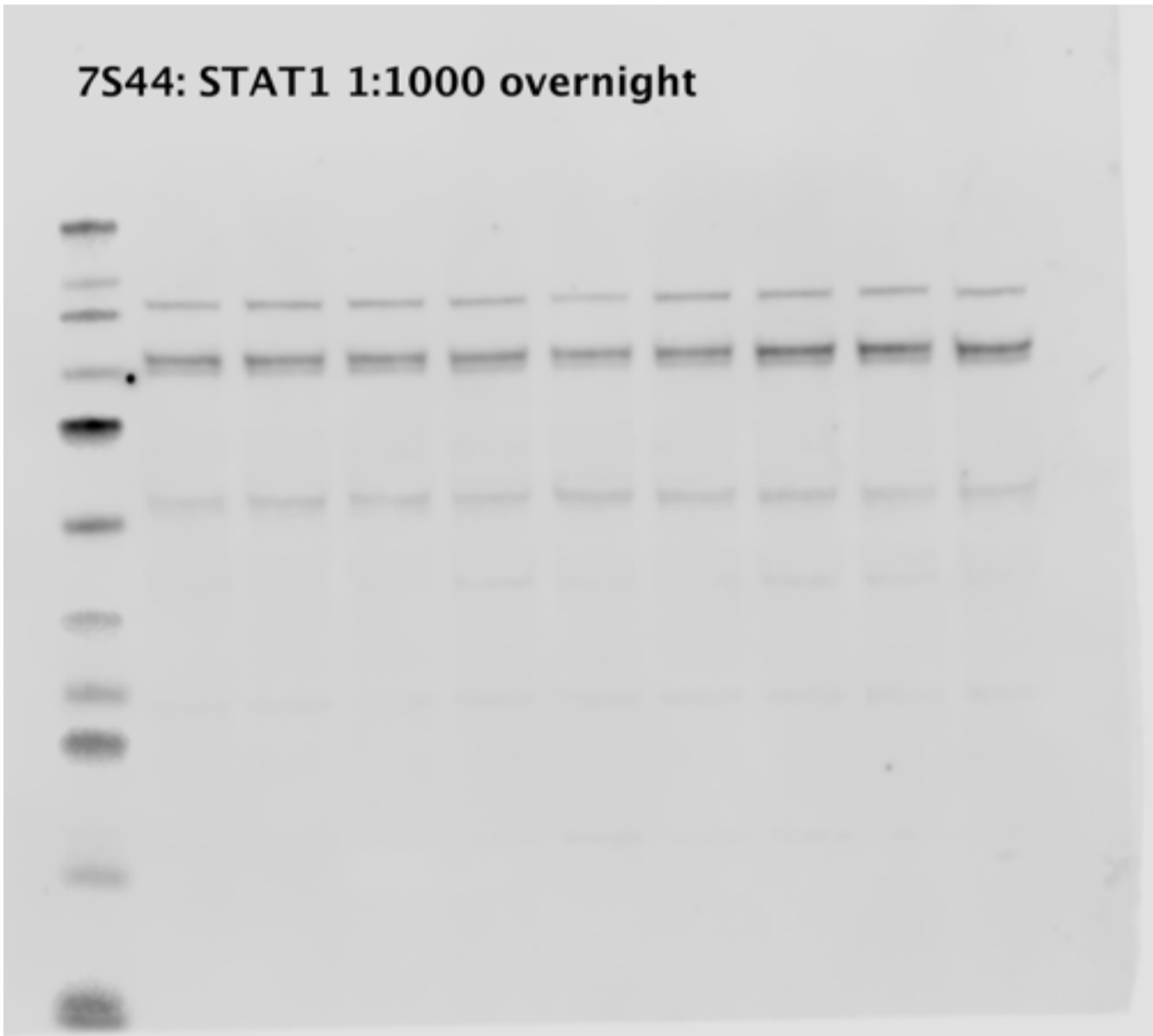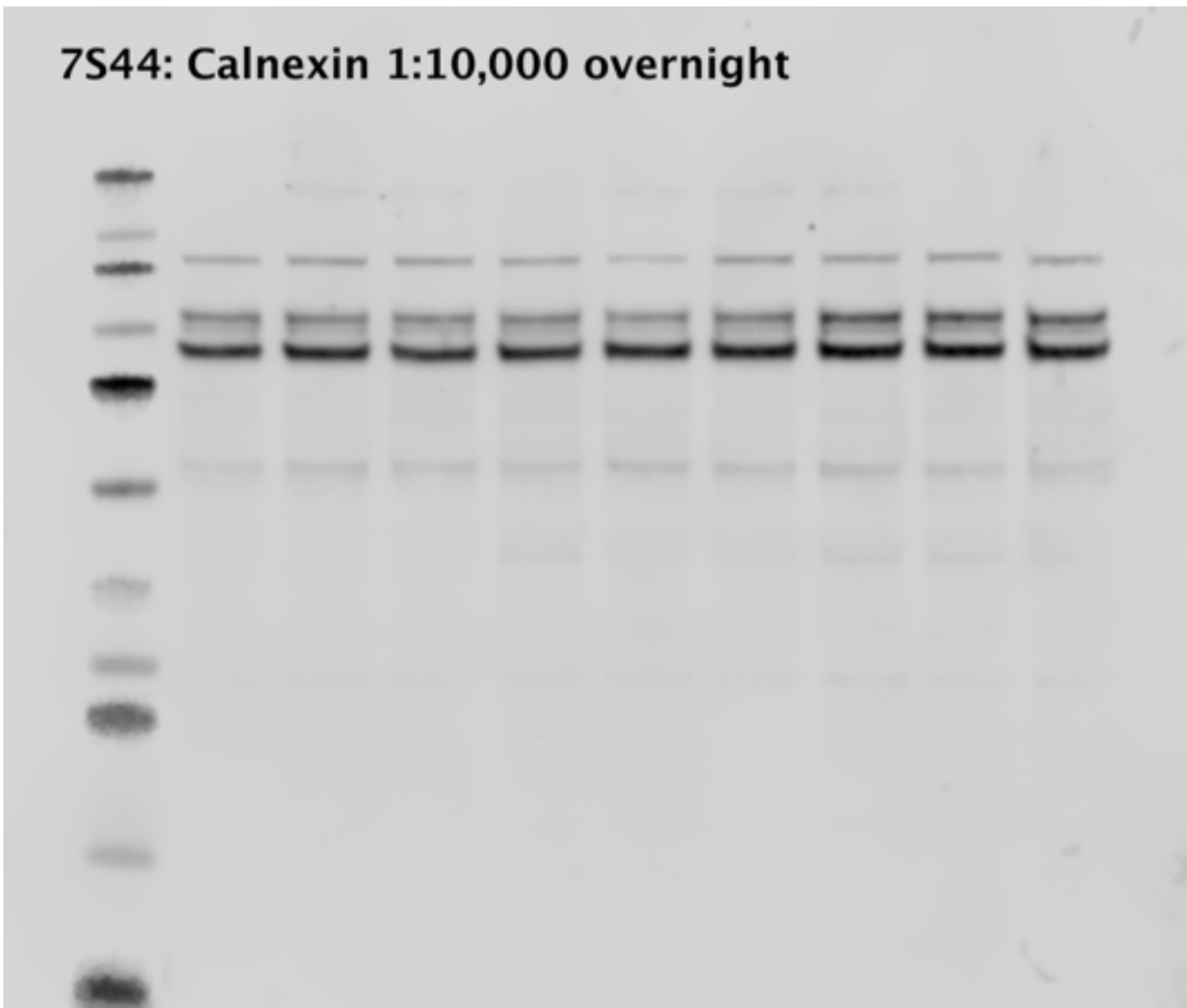

Supplement: Supplementary file 1 [file Data_Sheet_1.PDF]
