# Supplementary material for: Immunotolerant p50/NFκB Signaling and Attenuated Hepatic IFNβ Expression Increases Neonatal Sensitivity to Endotoxemia
Source: Front Immunol. 2018 Sep 26;9:2210. doi: 10.3389/fimmu.2018.02210 (PMC6168645; doi:10.3389/fimmu.2018.02210)
Supplement: Supplementary file 2 [file Data_Sheet_2.docx]

Supplementary Table 1:

| **Antibody** | **Vendor** | **Catalog Number** |
| --- | --- | --- |
| Anti-IFNβ | Cell Signaling Technology | 97450 |
| Anti-phospho-IRF3 | Cell Signaling Technology | 29047 |
| Anti-IRF3 | Cell Signaling Technology | 4302 |
| Anti-phospho-STAT1 | Cell Signaling Technology | 9167 |
| Anti-STAT1 | Cell Signaling Technology | 9172 |
| Anti-Lamin B | Santa Cruz Biotechnology | sc-6217 |
| Anti-HDAC1 | Cell Signaling Technology | 5356 |
| Anti-GAPDH | Cell Signaling Technology | 5174 |
| Anti-Calnexin | Enzo Life Sciences | ADI-SPA-860-D |
| Anti-MKP-5 | Santa Cruz | sc-374276 |
| Anti-PP2A C subunit | Cell Signaling Technology | 2259 |
| Anti-TBK1 | Cell Signaling Technology | 3504 |

Supplementary Table 2:

| **Target** | **Assay ID** |
| --- | --- |
| IFNβ | Mm001134790_g1 |
| IFIT1 | Mm00515153_m1 |
| IRG1 | Mm01224532_m1 |
| IKKε | Mm00444862_m1 |
| IP10 | Mm00445235_m1 |
| MCP1 | Mm00443258_m1 |
| IRF7 | Mm00516793_g1 |
| MDA5 | Mm00441242_m1 |
| OAS2 | Mm04207460_m1 |
| iNOS | Mm00459183_m1 |

**Supplementary Tables 1,2:** Immunoblotting Antibodies (Supplementary Table 1) and Real-Time qPCR primers (Supplementary Table 2).
